# Global phylogenomic analysis of Staphylococcus pseudintermedius reveals genomic and prophage diversity in multidrug-resistant lineages

**DOI:** 10.1099/mgen.0.001369

**Published:** 2025-03-05

**Authors:** Lucy F. Grist, Alice Brown, Noel Fitzpatrick, Giuseppina Mariano, Roberto M. La Ragione, Arnoud H. M. Van Vliet, Jai W. Mehat

**Affiliations:** 1 Department of Comparative Biomedical Sciences, School of Veterinary Medicine, Faculty of Health and Medical Sciences, University of Surrey, Guildford GU2 7A, UK; 2 MRC London Institute of Medical Sciences, Du Cane Road, London W12 0NN, UK; 3 Department of Life Sciences, Imperial College London, London SW7 2AZ, UK; 4 Department of Veterinary Clinical Sciences, School of Veterinary Medicine, Faculty of Health and Medical Sciences, University of Surrey, Guildford GU2 7AL, UK; 5 School of Infection and Immunity, University of Glasgow, Glasgow, UK; 6 Discipline of Microbes, Infection and Immunity, School of Biosciences, Faculty of Health and Medical Sciences, University of Surrey, Guildford GU2 7XH, UK

**Keywords:** phage defence, prophage, *pseudintermedius*, *Staphylococcus*

## Abstract

*Staphylococcus pseudintermedius* is the foremost cause of opportunistic canine skin and mucosal infections worldwide. Multidrug-resistant (MDR) and methicillin-resistant *Staphylococcus pseudintermedius* (MRSP) lineages have disseminated globally in the last decade and present significant treatment challenges. However, little is known regarding the factors that contribute to the success of MDR lineages. In this study, we compared the genome sequence of 110 UK isolates of *S. pseudintermedius* with 2166 genomes of *S. pseudintermedius* populations from different continents. A novel core genome multi-locus typing scheme was generated to allow large-scale, rapid and detailed analysis of *S. pseudintermedius* phylogenies and was used to show that the *S. pseudintermedius* population structure is broadly segregated into an MDR population and a non-MDR population. MRSP lineages are predicted to encode certain resistance genes either chromosomally or on plasmids, and this is associated with their MLST sequence type. A comparison of lineages most frequently implicated in disease, ST-45 and ST-71, with the phylogenetically related ST-496 lineage that has a comparatively low disease rate, revealed that ST-45 and ST-71 genomes encode distinct combinations of phage-defence systems and concurrently encode a high number of intact prophages. In contrast, ST-496 genomes encode a wider array of phage defence systems and lack intact and complete prophages. These findings indicate that MRSP lineages have significant structural genomic differences and that prophage integration and differential antiviral systems correlate with the emergence of successful genotypes.

**Impact Statement**

*Staphylococcus pseudintermedius* is a major cause of soft tissue infections in dogs but may occasionally infect other companion animals and humans, most often veterinary professionals in primary care. Methicillin-resistant *Staphylococcus pseudintermedius* (MRSP) have emerged worldwide and are often linked to resistance to multiple antimicrobials, resulting in a significant health burden. Here, we analysed a large collection of *S. pseudintermedius* genome sequences*,* which has allowed a detailed characterization of the molecular epidemiology and diversity of the species using a novel typing scheme.

Here, we show that closely related MRSP lineages differ in whether specific antibiotic resistance genes are encoded on potentially mobilizable plasmids, or more stably on the chromosome, indicating differing evolutionary trajectories of MRSP lineages. Comparison of the *S. pseudintermedius* types most frequently implicated in clinical cases to those associated with commensal carriage showed that known genes thought to contribute to disease are universal and, therefore, not associated with the high incidence rates of disease of particular lineages. We demonstrate that MRSP sequence types linked to high disease rates lack specific phage-defence systems and are associated with a high burden of prophages.

## Data Summary

All genome sequencing data (sequencing reads and genome assemblies) are available in the Sequence Read Archive and genome repositories at NCBI, in Bioproject number PRJNA1153484. The information for the individual *Staphylococcus pseudintermedius* genomes has been included in File S1, available in the online Supplementary Material. The genome assemblies used in this study are available at https://zenodo.org/records/13692319 (DOI: 10.5281/zenodo.13692318). The *S. pseudintermedius* cgMLST scheme developed for this study has been made available via Zenodo (https://zenodo.org/records/13633136; DOI: 10.5281/zenodo.13633135) and Figshare (https://doi.org/10.6084/m9.figshare.26911654.v1; DOI: 10.6084/m9.figshare.26911654). The epidemiological dataset has also been included as a Microreact project (https://microreact.org/project/fRMpbzQK8M8Dzq4tYnukSY-global-phylogenomic-analysis-of-staphylococcus-pseudintermedius-reveals-genomic-and-prophage-diversity-in-multi-drug-resistant-lineages), allowing for interactive analyses of our dataset and easy visualization of phylogenetic relatedness, disease and MRSP phenotypes of genomes from any country or continent.

## Introduction

*Staphylococcus pseudintermedius* is a common member of the skin and mucosal microflora of dogs and other companion animals [1]. However, it is also the foremost cause of opportunistic canine infections worldwide and is commonly implicated in pyoderma [2], otitis externa [3], post-operative bone infections and surgical abscesses [4,5] and respiratory and urogenital tract infections [4,6, 7]. The consequences of infection can range from mild tissue inflammation to severe necrosis [7]. Humans may play a role in transmission, particularly in reverse zoonotic transmission, as 4.1% of dog owners are estimated to be carriers of this organism [8,10]. Treatment of bacterial infections in companion animals often involves the same antimicrobial classes that are critical in human medicine, including first- and third-generation cephalosporins and fluoroquinolones [11]. Given the close proximity and contact between humans and companion animals, there is significant potential for the evolution and transmission of antimicrobial resistance in either host.

Recent genomic studies have shown a high level of genetic diversity in *S. pseudintermedius*, with more than 1400 multi-locus sequence types reported [12,14]. However, these studies have mostly primarily focused on singular geographic regions, leaving a picture of *S. pseudintermedius* lacking diversity. Moreover, epidemiological studies tend to focus on methicillin-resistant *Staphylococcus pseudintermedius* (MRSP) isolates of clinical relevance, which are often multidrug resistant (MDR). MDR and MRSP lineages have disseminated widely in the last two decades and present significant treatment challenges [2,3, 15]. However, this focus on MRSP isolates may distort the understanding of *S. pseudintermedius* species diversity and consequent understanding of other factors driving its evolution and emergence.

The most common sequence type implicated in *S. pseudintermedius* infections is ST-71, followed by ST-45 and ST-68 [12,13, 16,18]. Analysis of *S. pseudintermedius* populations in the USA has suggested that these MRSP genotypes arose through multiple independent gene acquisition events, including resistance genes, followed by clonal expansion [12,13], presumably facilitated by the use of many classes of antibiotics in veterinary medicine. MRSP isolates have been reported to encode a larger accessory genome than their MSSP counterparts [19]. This suggests that gene acquisition is a primary driver in the emergence of pathogenicity.

The prevalence of genes associated with antibiotic resistance, virulence, prophages and horizontal gene transfer (HGT) has been reported to differ amongst the epidemic clones [14], and epidemiological studies clearly indicate that resistance to antimicrobials is not a pre-requisite for disease, as evidenced by antibiotic-sensitive isolates implicated in disease [17]. Identifying the characteristics that dictate the dominance of certain genotypes in clinical cases, such as ST-71 and ST-45, over other disease-causing * S. pseudintermedius* lineages is required to understand patterns of dissemination and improve surveillance.

In this study, we have aimed to identify the factors that contribute to the success of genotypes associated with dissemination and clinical disease beyond the acquisition of resistance genes. To allow the comparison and clustering of a large number of *S. pseudintermedius* genomes, we generated a core-genome MLST scheme. We then used this new scheme to characterize the diversity of the *S. pseudintermedius* phylogeny and identified that MDR/MRSP lineages differ in whether specific antibiotic resistance genes are encoded on plasmids or the chromosome. Furthermore, we show that sequence types associated with multi-continent dissemination and high rates of clinical disease encode a high density of prophages and a distinct combination of phage defence systems.

## Methods

### Collection, culture and sequencing of *S. pseudintermedius* clinical isolates from the UK

We performed whole-genome sequencing on 110 *s*. *pseudintermedius* isolates collected from a single veterinary referral practice between 2015 and 2017. All isolates were obtained from canine patients as part of routine clinical practice and confirmed as * S. pseudintermedius* by loop-mediated isothermal amplification (LAMP) and culture [5]. Individual isolates were cultured from cryovials stored at −80 °C onto Columbia blood agar (Oxoid, UK), aerobically at 37 °C for 24 h. DNA was extracted using the GenElute Bacterial Genomic DNA Kit (Sigma, UK). Genome sequencing was provided by MicrobesNG (http://www.microbesng.com). Genomic DNA libraries were prepared using the Nextera XT Library Prep Kit (Illumina, San Diego, USA), following the manufacturer’s protocol with the following modifications: input DNA was increased twofold, and PCR elongation time was increased to 45 s. DNA quantification and library preparation were carried out on a Hamilton Microlab STAR automated liquid handling system (Hamilton Bonaduz AG, Switzerland). Libraries were sequenced using Illumina sequencers (HiSeq) with a 250 bp paired-end protocol. Sequence adapters were trimmed using Trimmomatic (version 0.30) with a sliding cut-off of Q15 and assembled using Shovill version 1.1.0 (https://github.com/tseemann/shovill) using the default settings and the SPAdes assembler [20].

### Download of publicly available *S. pseudintermedius* genomes and associated metadata

Metadata for all available *S. pseudintermedius* genomes were downloaded from the NCBI Pathogens Detection database (https://www.ncbi.nlm.nih.gov/pathogens/). Corresponding assemblies were downloaded and quality-assessed using QUAST version 4.6.3 [21]. Samples for which only sequence reads were available (File S1) were processed for assembly with Shovill/SPAdes as above. Only genomes with N50 >50 kb, L50 <20 and a number of contigs <200 were included in this study. Assemblies with an absence of information on the isolation source were excluded. The final dataset of 2276 *s*. *pseudintermedius* genomes (File S1) consisted of 110 UK genomes and 2166 publicly available sequences representing the wider geographical population. Each assembly was categorized as disease or non-disease, according to metadata. Samples derived from pyoderma, wounds, abscesses or internal organs were classed as ‘disease’, whereas samples isolated from the skin of healthy hosts were classed as ‘non-disease’.

### Phylogenetic reconstruction, multi-locus sequence typing and core-genome sequence typing

The UK assemblies were typed according to the PubMLST scheme for *S. pseudintermedius* (https://pubmlst.org/organisms/staphylococcus-pseudintermedius) using MLST version 2.23.0 (https://github.com/tseemann/mlst). Phylogenetic reconstruction of UK *S. pseudintermedius* genomes was performed based on core-genome SNPs using ParSNP version 1.7.4 [22]. Due to a high degree of genotypic variation, a core-genome MLST scheme was developed using chewBBACA version 2.8.5 [23] with default settings (https://github.com/B-UMMI/chewBBACA_tutorial). A training file was generated using Prodigal version 2.6.3 [24]. The scheme was generated using 74 complete genomes and consists of 1356 genes present in 99% of the genomes. cgMLST types were designated relative to conventional MLST sequence types. Phylogenetic trees from the chewBBACA allele calls were constructed using GrapeTree version 1.5.0 and the RapidNJ algorithm [25]. The *S. pseudintermedius* cgMLST scheme is available from Zenodo (DOI: 10.5281/zenodo.13633135) and Figshare (DOI: 10.6084/m9.figshare.26911654).

### Detection of virulence and antimicrobial resistance genes and prediction of plasmid contigs

The NCBI AMRFinderPlus tool version 3.10 [26] was used to identify antimicrobial resistance genes and resistance-associated point mutations, using the default settings. AMRfinder information was used to assign each genome as either MRSP (*mecA*-positive) or MSSP (*mecA*-negative). Abricate version 1.0.1 (https://github.com/tseemann/abricate) was used to search for known *S. pseudintermedius* virulence genes [17] and biocide resistance genes in all genomes, using a 90% coverage and 90% identity threshold. Prediction of plasmid and chromosomal contigs was performed using RFplasmid [27] using the provided *Staphylococcus* analysis file. Contigs with a >50% probability score were considered to be of plasmid origin.

### Pangenome-wide analysis for markers enriched in disease-associated backgrounds

All genome assemblies were annotated using Prokka version 1.14.6 [28]. A pan-genome was constructed using Roary version 3.13 [29] with a blast cut-off of 95%. Gene markers over-represented in genotypes associated with high clinical disease (e.g. ST-71, ST-45 and ST-258) and low clinical disease (e.g. ST-496) within a narrow genetic background were identified using Scoary version 1.16 [30] with an initial threshold of a Bonferroni-corrected *P*-value of 0.05. Genes determined to be present in >90% of the target genomes and in <10% of the non-target genomes were considered significant. Selected genomes associated with a high density of putative phage genes were screened using PHASTEST [31], confirming the presence of prophages. Putative phage defence systems and genes were identified using PADLOC v2.0.0 PADLOC-DB v2.0.0 with default settings [32]. Prophages SPpB and SPpC have been annotated using Pharokka [33], and schematics of the phage genes are included in the Supplementary Material (Figs S7 and S8).

## Results

### Population structure of *S. pseudintermedius* isolated in the UK

**Fig. 1.**
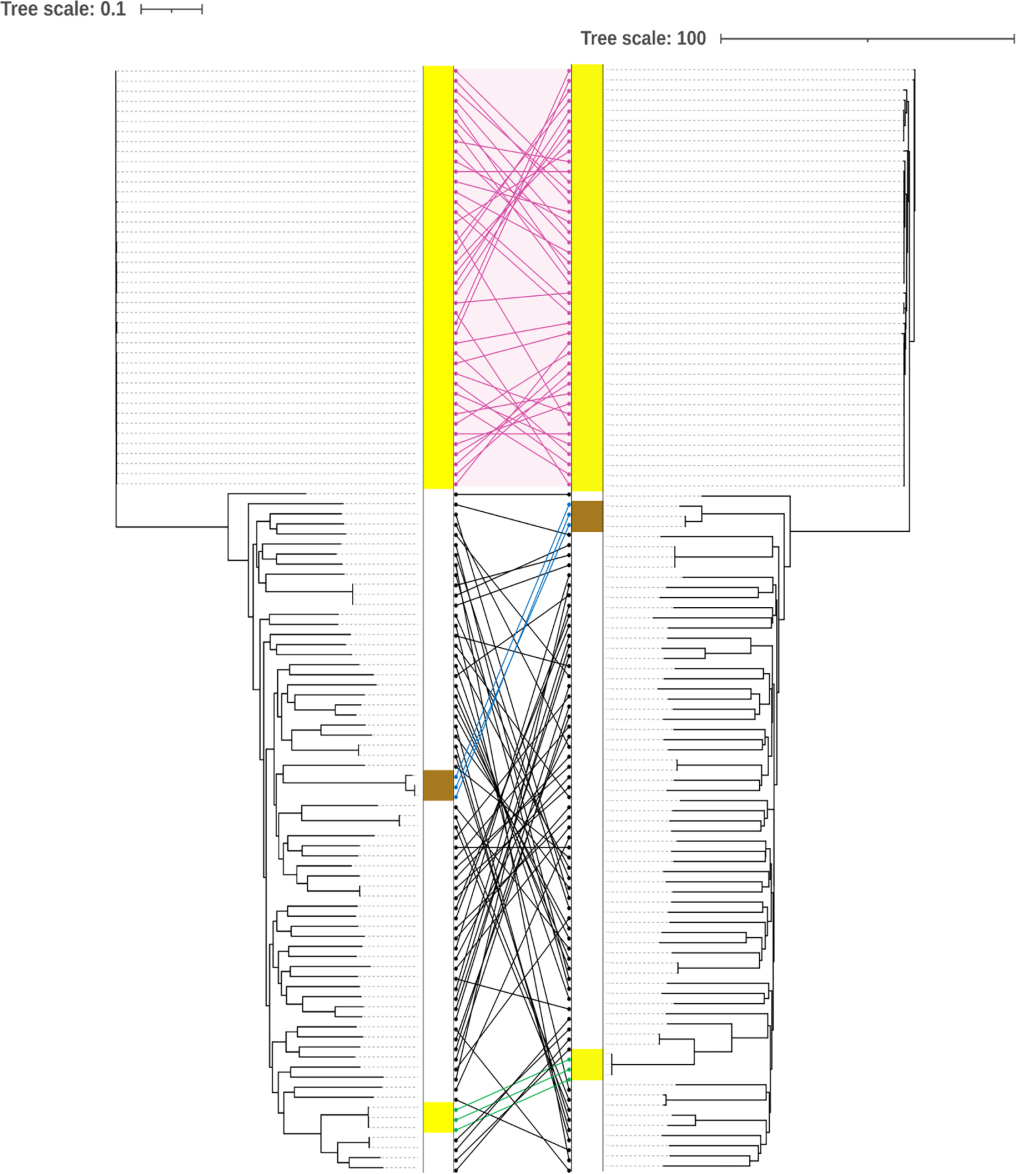
Tanglegram showing a comparison of phylogenetic reconstruction of 110 S. *pseudintermedius* genomes collected in this study from clinical cases in the UK as part of routine clinical practice. The tanglegram shows a comparison of core-genome SNPs using ParSNP (left) and a reconstruction based on cgMLST using GrapeTree (right). Phylogenetic analysis based on the cgMLST scheme correlated well with the existing pubMLST scheme, and clustering of major clonal complexes, such as ST-71 (pink shading and connecting lines), is largely conserved.

Within a UK veterinary referral practice, *S. pseudintermedius* was implicated in ~30% of all bacterial infections arising from surgical site infections over a 4-year period. A total of 110 isolates were obtained, and their genome sequence was determined, which was then used to analyse their population structure. Within these 110 isolates, ST-71 was the dominant sequence type, accounting for ~40% of the *S. pseudintermedius* isolates in the collection. Forty-nine isolates derived from clinical *S. pseudintermedius* infections in the UK were unable to be assigned a sequence type according to the existing MLST scheme [34]. These novel clonal complexes were composed of highly diverse genotypes indicative of a highly varied population structure of disease-causing isolates in the UK, beyond the dominant ST-71 lineage (Fig. 1).

A total of 57.2% of our UK clinical isolates were classified as MDR, and 51% were determined to be MRSP. Beyond ST-71, the MDR/MRSP trait was also found in disparate MLST types, including ST-45, ST-277 and ST-301. To more accurately characterize and interrogate the dataset, a core-genome MLST scheme was generated and implemented, which allowed discriminative typing of all genomes. Phylogenetic analysis based on the cgMLST scheme correlated well with the existing pubMLST scheme and phylogenetic typing based on core-genome SNPs (Fig. 1), and as cgMLST allows for the analysis of larger genome data sets, all subsequent phylogenetic analyses were based on cgMLST.

### Pan-genome analysis of *S. pseudintermedius*

An additional 2166 *s*. *pseudintermedius* genomes acquired from the NCBI Pathogens Detection database were acquired to supplement the UK *S. pseudintermedius* dataset and construct a pan-genome more representative of *S. pseudintermedius* genomes circulating worldwide (File S1). Virulence gene and antimicrobial resistance gene distribution within the 2276 *s*. *pseudintermedius* genomes were determined within the context of associated metadata for each genome. Genomes were classified as MDR if intact resistance genes to three or more different classes of antibiotics were detected. The major MDR genotypes were determined, and the correlation of MDR with disease and geographical site of isolation was evaluated.

Of the 8809 unique genes within the pan-genome, only 1995 were classified as core genes. Remarkably, of the 6814 accessory genes identified, 6099 were present in only 0–15% of genomes. This suggests that the majority of accessory genes are lineage-specific, or even unique to single isolates, and are not shared amongst all genotypes, despite occupying the same niche. Consistent with this, the rate at which novel accessory genes were detected remained constant as genomes were incorporated into the pan-genome (Fig. S1), indicating that *S. pseudintermedius* has an open pan-genome characterized by high phylogenetic diversity and acquisition of novel genes through horizontal transfer. This suggests that the acquisition of novel genetic material is widespread in the species and may profoundly impact the population structure.

### Epidemiology, clinical disease association and multidrug resistance of *S. pseudintermedius* genotypes

**Fig. 2.**
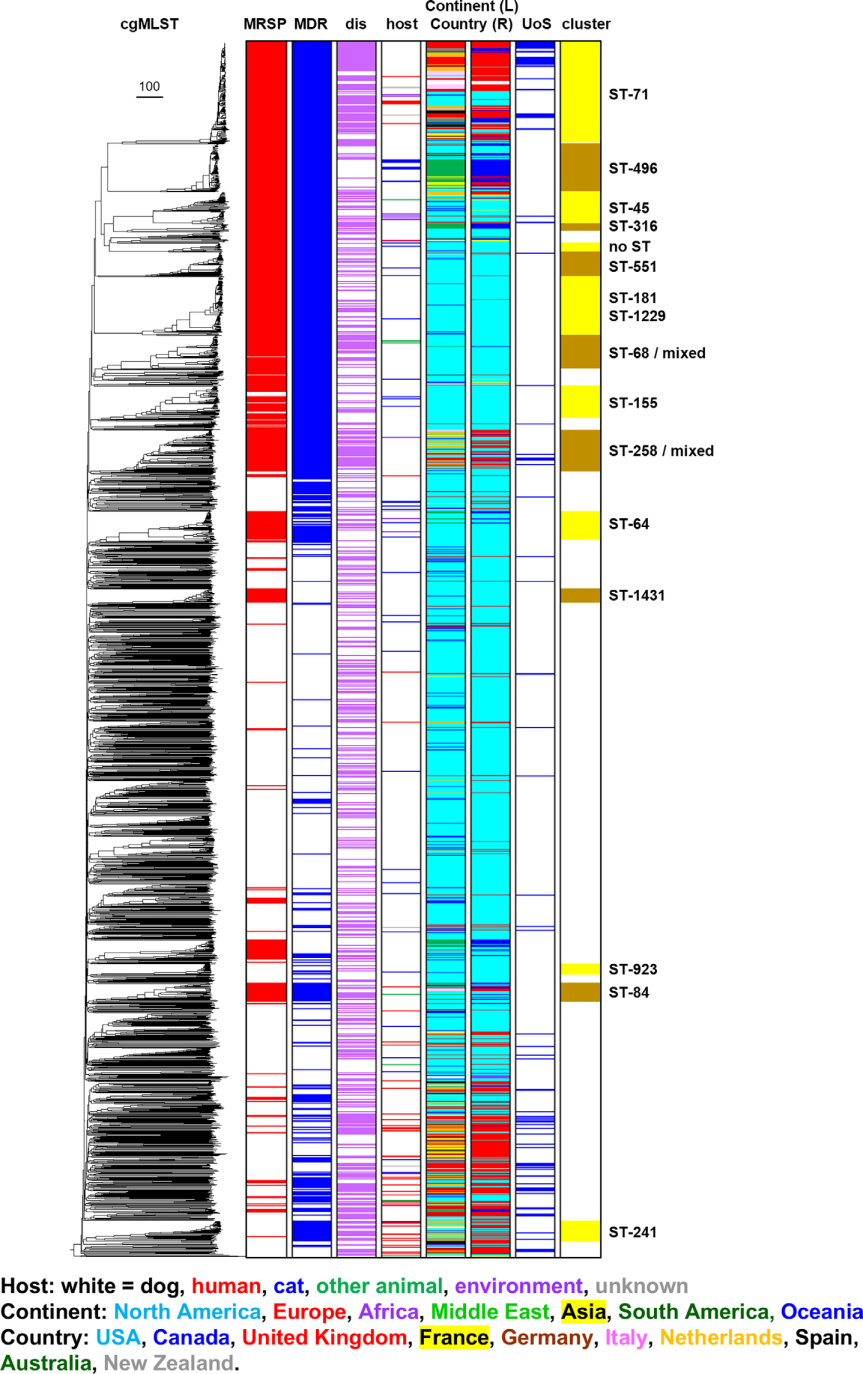
cgMLST phylogeny of 2276 *S. pseudintermedius* assembled genomes (File S1) consists of a range of commensal and disease-causing isolates (purple shading, column labelled ‘dis’), predominantly originally isolated from dogs (white shading, column labelled ‘host’). The dataset encompasses genomes derived from multiple continents: North America, Europe, Africa, Middle East, Asia, South America and Oceania. Genomes sequenced as part of this study are coloured blue in the UoS column, and publicly available genomes from sequence repositories are white in this column. Genomes of isolates classed MDR on the basis of encoding genes conferring resistance against three or more classes of antibiotics are shaded blue in the column labelled ‘MDR’. Genome classes as MRSP are coloured red in the ‘MRSP’ column. The ‘cluster’ column refers to the cgMLST phylogenetic cluster; MDR/MRSP phylogenetic clusters are predominantly composed of ST-71, ST-496, ST-45, ST-316, ST-551, ST-181, ST-1229, ST-68, ST-155 and ST-258. The ‘mixed’ label is included in the ST-68 and ST-258 phylogenetic cluster labels as these clusters are composed of genomes that are not only predominantly classified as ST-68 and ST-258, respectively, but also encompass closely related genomes that differ in one or more core-genome alleles, and are therefore given a different cgMLST designation.

The 2276 *s*. *pseudintermedius* genomes comprise a range of commensal and disease-causing isolates. However, the dataset is heavily skewed towards genomes derived from the USA; consequently, there are many genotypes that appear to be geographically restricted, particularly within the MDR cluster, for example, ST-155, ST-64 and ST-551. In contrast, there are clonal complexes that are widely disseminated, including ST-71, ST-45 and ST-258, and to a lesser extent ST-496, which are also MDR lineages (Fig. 2).

**Fig. 3.**
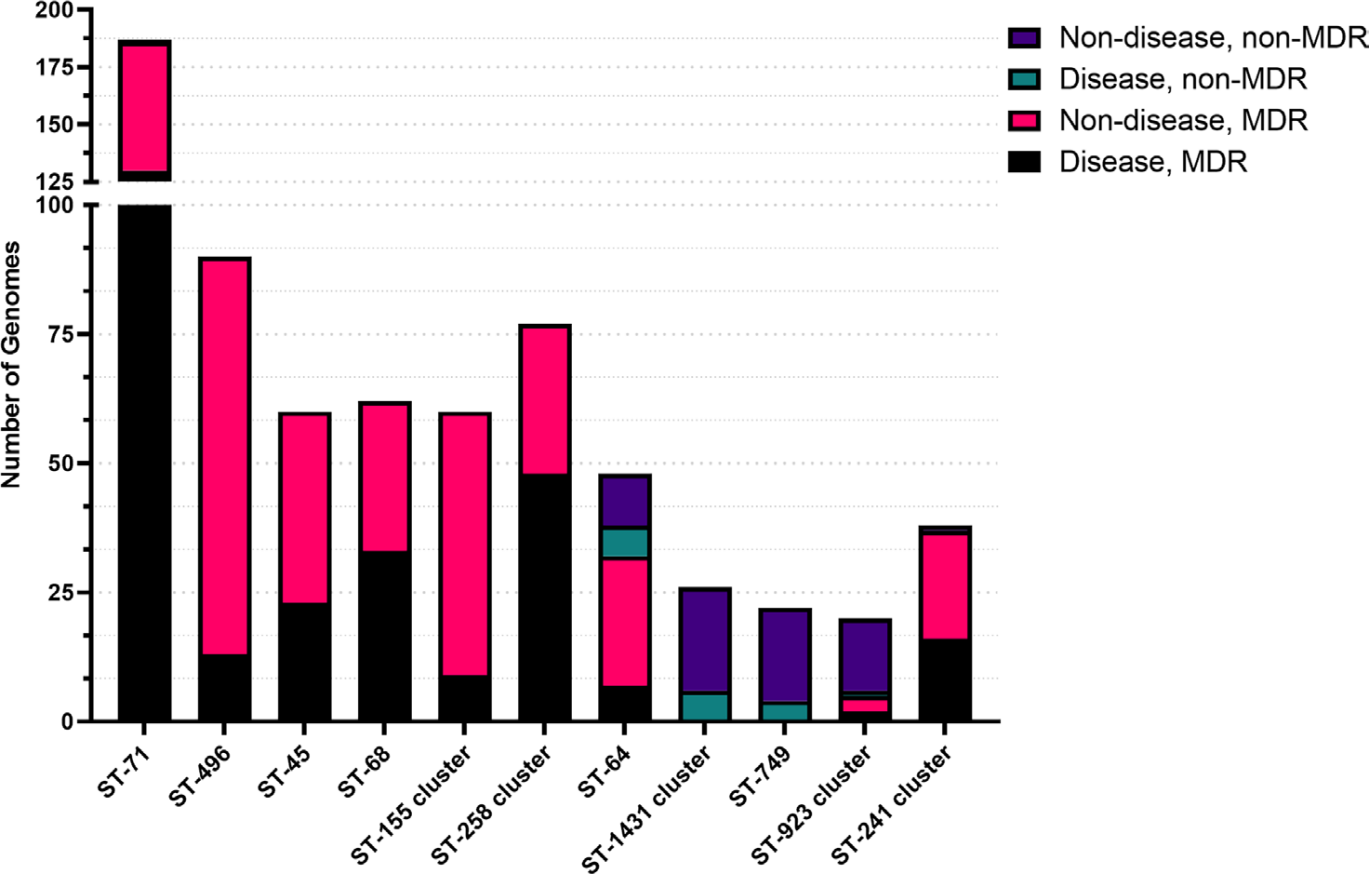
Stacked bar plot showing *S. pseudintermedius* sequence type association with disease and MDR status. ST-71 is an MDR lineage that is strongly associated with disease. Conversely, ST-496 is a closely related MDR sequence type that is associated with commensal carriage. ST-1431, ST-749 and ST923 are non-MDR lineages that are more commonly associated with commensal carriage.

Almost one-third (32%) of the *S. pseudintermedius* genomes in the whole dataset are associated with clinical disease (Table 1). However, certain clonal complexes were over-represented in this disease-associated group (Fig. 3; Table 2). More than two-thirds (70%) of ST-71 genomes are associated with clinical disease. Conversely, ST-496, which is phylogenetically closest to ST-71, consists of genomes that are primarily derived from non-diseased hosts (86%), indicating that this clonal complex is strongly associated with commensal carriage.

**Table 1.** The proportion of 2274 *S*. *pseudintermedius* genomes used in this study that are classified as either MRSP or MSSP and their association with commensal carriage (non-disease) or clinical (disease) cases

MRSP/MSSP	Non-disease	Disease	Total
MRSP	569	389	958 (42%)
MSSP	978	340	1318 (58%)
**Total**	1547 (68%)	729 (32%)	**2276**

**Table 2.** A breakdown of prevalent *S. pseudintermedius* sequence types and their association with MDR status and rates of clinical cases (disease)

Sequence type	MDR	Non-MDR	Non-disease	Disease	Total no. of genomes
ST-71	86	1	56	131	**187**
ST-496	90	0	77	13	**90**
ST-45	60	0	37	23	**60**
ST-68	21	0	5	16	**21**
ST-155	36	0	34	2	**36**
ST-258	23	0	9	14	**23**
ST-64	32	16	35	13	**48**
ST-1431	0	19	15	4	**19**
ST-749	0	21	17	4	**21**
ST-923	4	14	15	3	**18**
ST-241	22	0	11	11	**22**

Of the genomes derived from clinical samples, 51% (424/729) are classified as MDR. Of the non-clinical samples, 45% (699/1547) were classified as MDR. There appears to be no combination of resistance genes that is more associated with either disease or non-disease genomes. This shows that MDR status is not a pathogenic trait in itself and is not a predictor of clinical disease.

**Fig. 4.**
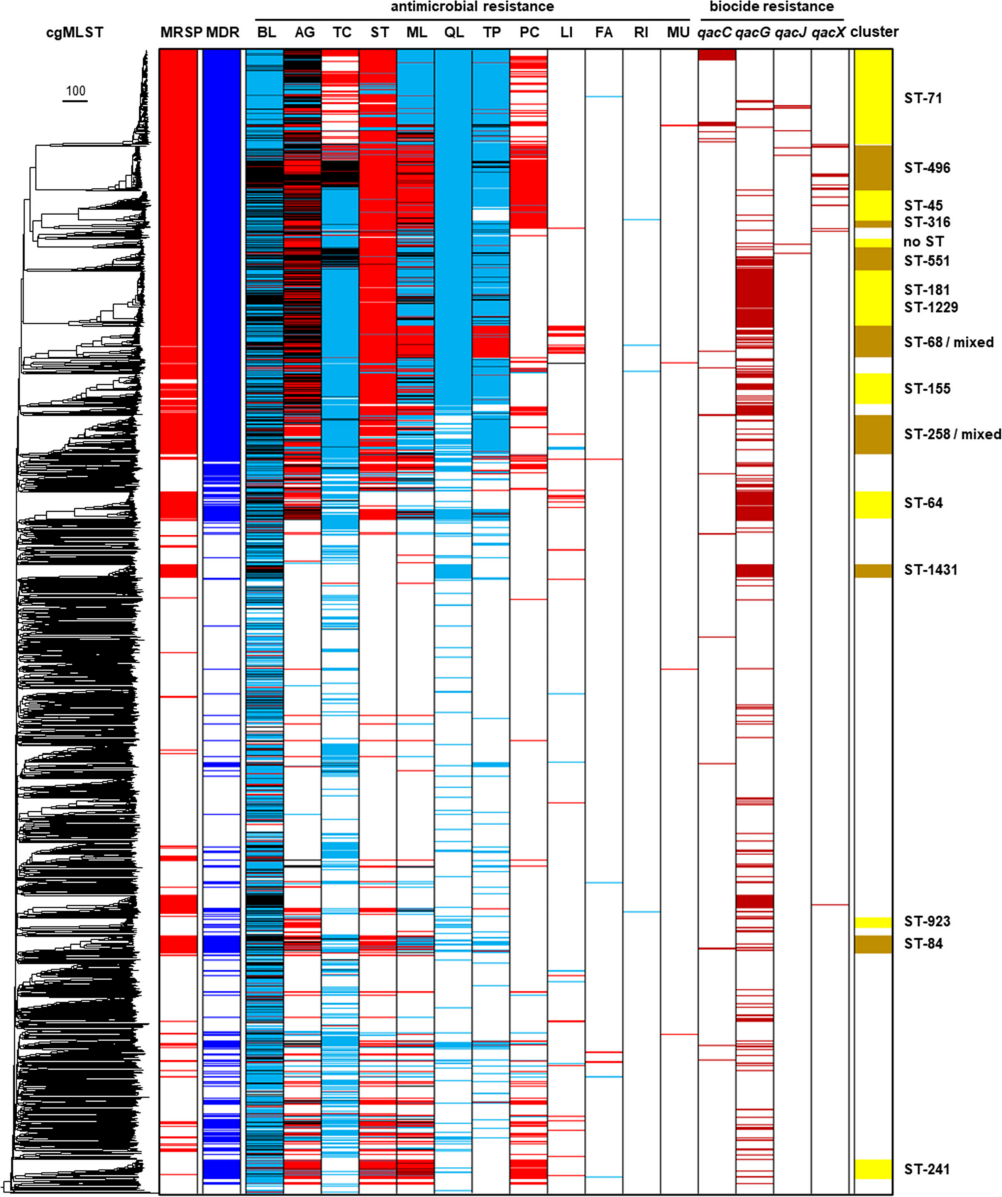
The phylogenetic distribution of antimicrobial resistance (AG, aminoglycoside; BL, beta-lactam; FA, fusidic acid; LI, lincosamide; ML, macrolide; MU, mupirocin; PC, phenicol; QL, quinolone; RI, rifampicin; ST, streptothricin; TC, tetracycline; TP, trimethoprim) and biocide (*qacC, qacG, qacJ* and *qacX*) genes in *S. pseudintermedius*. The ‘cluster’ column refers to the cgMLST phylogenetic cluster. The ‘mixed’ label is included in the ST-68 and ST-258 phylogenetic cluster labels as these clusters are not only composed of genomes that predominantly are classified as ST-68 and ST-258, respectively, but also encompass closely related genomes that differ in one or more core-genome alleles, and are therefore given a different cgMLST designation. Each gene was predicted to be encoded on either the chromosome (blue shading), plasmid (red shading) or both (black shading). Sequence types differ in the manner by which resistance genes are encoded; tetracycline resistance is chromosomally encoded across the phylogeny except in ST-71, where it is encoded on a plasmid. Trimethoprim resistance is encoded chromosomally by MDR lineages, with the exception of ST-68. The ST-496, ST-45, ST-316 and ST-68 clusters encoded macrolide resistance on predicted plasmid contigs, whereas most other MDR lineages are predicted to encode macrolide resistance on the chromosome. Full details of the phylogenetic distribution of antimicrobial resistance and biocide genes are provided in File S1.

The *S. pseudintermedius* phylogeny can be broadly segregated into an MDR population and a non-MDR population. Each contig from the 2276 assemblies in this collection was classified as either plasmidic or chromosomal based on the Rfplasmid score; this revealed that the MDR population varies significantly in how resistance genes are encoded (Fig. 4). Beta-lactamase resistance is encoded not only across the phylogeny, most often on the chromosome, but also on both the chromosome and a plasmid, as in the case of an ST-496 sub-population. Aminoglycoside resistance is associated with the MDR population and is encoded by plasmids or both a plasmid and on the chromosome. Tetracycline resistance is chromosomally encoded across the phylogeny except in ST-71, where it is encoded on a plasmid. ST-496 and ST-551 encode tetracycline resistance on both the chromosome and a plasmid. The ST-496, ST-45, ST-316 and ST-68 clusters encoded macrolide resistance on predicted plasmid contigs, whereas most other MDR lineages are predicted to encode macrolide resistance on the chromosome. Similarly, trimethoprim resistance is encoded chromosomally by MDR lineages, with the exception of ST-68, which encodes this resistance on predicted plasmid contigs. The genes, mutations and alleles conferring predicted resistance to the antibiotic classes outlined above are detailed in File S1.

Virulence gene content showed no association with disease outcome and was not different between major lineages (Fig. S2), suggesting that all genotypes are capable of causing opportunistic disease via intrinsic mechanisms encoded across the phylogeny. Therefore, virulence gene density cannot account for the dominance of ST-71, ST-68 and ST-258 relative to other genotypes. In light of this, we sought to identify genetic factors that contribute to the success of ST-71 and ST-45, beyond their MDR/MRSP status.

### Prophages are major determinants of *S. pseudintermedius* diversity and reflect the distribution of phage defence systems

Genome-wide association studies were performed to identify genetic markers that are enriched (>95%) in phylogenomic clusters within the MDR phylogenetic cluster associated with multiple country incidence and a high degree of clinical disease, such as the genotypes ST-71 and ST-45 (Table S1). Concurrently, we used this approach to identify markers associated with genotypes with a comparatively lower rate of clinical disease such as in ST496. We determined the distribution of these markers across the phylogeny and assessed their impact on the population structure. Many genes over-represented in ST-71 and ST-45 were annotated as phage genes (Table S1). We used PHASTEST to characterize these putative prophages (Figs S4-S6).

**Fig. 5.**
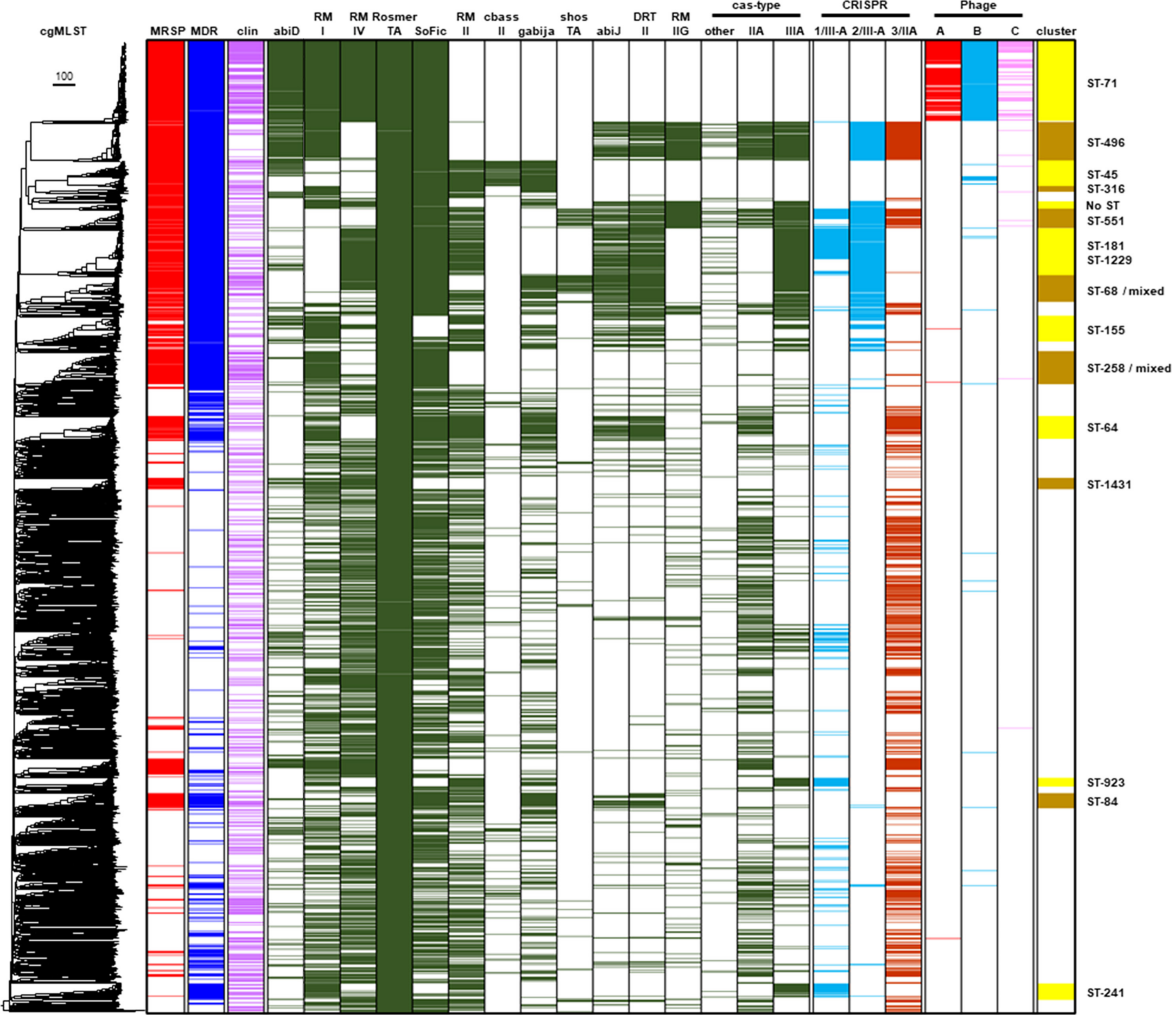
The combination of phage defence systems encoded by *S. pseudintermedius* reflects the density of prophages detected in the genomes. All 2276 genomes were screened for the presence of specific prophages detected by PHASTEST, and PADLOC was used to detect antiviral genes and systems (abiD=abortive infection system protein D, RM I=type I restriction-modification system, RM IV=type IV restriction-modification system, RosmerTA=RosmerTA system encoded by *rmrT* WP_231741552.1 and *rmrA* WP_058719324.1, SoFIC=SoFIC antiviral gene WP_242883646.1, RM II=type II restriction-modification system, cbass II=CBASS operon composed of four genes, Gabija=Gabija phage defence system composed of GajA and GajB, shoshTA=shoshTA toxin/antitoxin system, abiJ=Abortive infection system protein J, DRT II=type II defence-associated reverse transcriptase, RM IIG=type IIG restriction-modification system, cas-type/CRISPR=CRISPR Cas systems). The ‘cluster’ column refers to cgMLST phylogenetic cluster. The ‘mixed’ label is included in the ST-68 and ST-258 phylogenetic cluster labels as these clusters are composed of genomes that are not only predominantly classified as ST-68 and ST-258, respectively, but also encompass closely related genomes that differ in one or more core-genome alleles, and are therefore given a different cgMLST designation. ST-71 encodes SpST71A, SPpB and SPpC prophages [columns labelled Phages A (red), B (blue) and C (pink)], which are largely absent from other lineages. ST-71 also encodes a distinct combination of phage defence genes (indicated by green) relative to most other lineages. ST- 496, which has a dearth of prophage sequences, encodes a unique combination of antiviral genes. Full details of the phylogenetic distribution of identified phage defence systems and prophages are provided in File S1.

Three specific prophage sequences were primarily associated with ST-71, ST-45 and ST-258, the lineages most frequently implicated in clinical disease (Fig. 5), including the SPST71A prophage that has previously been linked to the disruption of genetic competence by inactivation of the *comG* gene [14]. Another of these prophages, designated here as SPpB, is 28.1 kb in length and is chromosomally integrated in ST-71, and in ST-45 adjacent to core genes involved in cell division (*whiA*) and metabolism (Fig. S4). There is a large (2 kb) intergenic space upstream of the SPpB integrase and upstream of the start codon on the antisense strand, so it is unlikely that SPpB integration at this site has influenced the transcription of adjacent genes.

An additional 43.7 kb complete prophage associated with certain MRSP backgrounds was identified. This prophage, designated here as SPpC, appears to have integrated into the genomes of *S. pseudintermedius* ST-71, but exhibits variation in ST-45 and ST-68, and is significantly truncated in other genetic backgrounds (Fig. 5). SPpC is inserted adjacent to the core genes, including the *suf* operon involved in oxidative stress repair, and *corB* involved in Mg^2+^ and Co^2+^ transport, as well as tRNA genes, which are common targets for mobile genetic elements in bacterial genomes [35]. In-depth annotation of these prophages is shown in (Figs S7 and S8) . Of the 233 ST-71 and ST-45 genomes, 153 (66%) encode SPST71A, 198 (85%) encode SPpB and 71 (30%) encode SPpC, demonstrating that the presence or absence of prophages accounts for variation in these genomes.

In contrast to related clonal complexes, such as ST-71 and ST-45, intact prophages were not detected in ST-496. Seeking an explanation for the lack of intact prophages in this lineage, GWAS determined that ST-496 encodes two independent type IIIA CRISPR-Cas systems absent from ST-71 and ST-45. Following this, we used PADLOC to systematically detect known anti-phage systems across the phylogeny and found that the repertoire of phage defence systems encoded differs markedly between these closely related lineages. ST-71 and ST-45 encode fewer phage defence systems than any other lineage (Item S1). Furthermore, the combination of the antiviral genes they do possess is distinct relative to other MDR-associated sequence types. ST-71 encodes the abortive infection system AbiS, two restriction-modification (RM) systems (types I and IV) and SoFic, in addition to the RosmerTA defence system encoded by all *S. pseudintermedius* (Fig. 5). ST-496 lacks the type IV RM system, but also encodes type IIA and type IIIA CRISPR-Cas systems, AbiJ, DRT class II and a type IIG RM system. ST-45, on the other hand, predominantly encodes a CBASS type IIs, type II RM system and Gabija, as well as the occasional instance of either AbiD or a type I RM system. The variation in the number and type of phage defence systems encoded by these related lineages reflects the presence and absence of SpST71A, SPpB and SPpC and is consistent with a scenario, wherein the combination of defence systems encoded by ST-496 confers stronger resistance to prophage insertion.

## Discussion

In this study, we present a comprehensive population genomic analysis of *S. pseudintermedius* using a novel core-genome multi-locus sequence typing scheme, which has facilitated the classification of novel genotypes. The *S. pseudintermedius* phylogeny is broadly segregated into an MDR/MRSP population and a non-MDR/MRSP population, indicating that despite shared niche access to a variety of mobile antimicrobial resistance genes, carriage of certain resistance genes appears to be restricted to select lineages. Within the MDR/MRSP population, we have identified MRSP lineages that exhibit differences in the means by which select resistance genes are encoded, either chromosomally or on plasmids. This indicates a degree of genomic variation within this population that is linked to genetic background.

Consistent with previous reports [14,15, 17], our results indicate the co-circulation of a diverse range of lineages and an open pan-genome consistent with the accumulation of genetic material via HGT. One important mechanism of HGT is prophage acquisition, wherein phage genomes integrate into the bacterial chromosome. Our results suggest that prophage integration between otherwise closely related MRSP lineages is a feature of ST-71, ST-45 and ST-258 and is correlated with higher disease rates. Within this MDR population, the absence of intact prophages in ST-496, which is associated with commensal carriage, is likely due to a unique array of phage defence systems, which may have rendered ST-496 resistant to phage infection and lysogenic carriage of SpST71A [14], SPpB and SPpC. Considering prophages represent the major genome divergences between these lineages, the differences in the antiviral arsenal encoded by specific lineages appear to have had a significant impact on the evolution of MRSP.

Aside from contributing to genetic diversity, bacteriophages often impact phenotypically on their host as observed in *Staphylococcus aureus*, wherein bacteriophages and other mobile genetic elements mediate the transfer of pathogenicity islands, conferring new phenotypic traits that enable bacterial adaption [36]. For example, the Sa3int phages, which are the most prevalent of * S. aureus* phages, carry the immune evasion cluster, which encodes immunomodulatory proteins Sak, Scin and CHIPS, which act in concert to enable within-host survival [37,38].

Prophage carriage is also associated with bacterial virulence in many other species; for example, scarlet fever is caused by specific strains of *Streptococcus pyogenes* that encode a phage-derived toxin; only *S. pyogenes* that are lysogenic for these phages can cause scarlet fever [39]. Similarly, in *Corynebacterium diptheriae,* its main virulence factor, diphtheria toxin, is encoded on a prophage [40]. *Escherichia coli* O157:H7 and O104:H4 have acquired the Shiga toxin-encoding gene *stx2a,* through lysogeny [41]. Investigating the impact of prophage sequences identified in this study on *S. pseudintermedius* pathobiology is an attractive future direction for *S. pseudintermedius* studies.

The large prophages, SPpB and SPpC, which have integrated into ST-71, ST-45 and ST-68 isolates, may contribute to the high disease rate associated with these genetic backgrounds, relative to ST-496, which does not encode these prophages. Whilst * S. pseudintermedius* prophages themselves may harbour, and co-select for, genes that contribute to the high rate of disease caused by ST-71 and ST-258, the site of phage integration in the genome may also play a role. Integration of prophages into core gene regions has been shown to have an impact on expression levels of adjacent genes as observed previously [42,45] and can consequently be expected to contribute to physiological changes. This is exemplified by the SpST71A prophage unique to ST-71, which is inserted into the *comG* gene and is suspected to disrupt the natural genetic competence of this lineage [14]. The quiescent prophages identified in this study do not appear to have interrupted any identifiable gene but are adjacent to core genes involved in cell division, oxidative stress repair and metal transport. The integration and expression of prophages is a complex series of interactions that involve pirating host cell transcriptional machinery [46], which may impact downstream and upstream genes.

Ultimately, it is clear that multiple phage integration events have affected genome architecture in the different lineages, which can affect phenotypic variation. The maintenance of multiple intact prophages in ST-71, 45 and 258 genomes compared with closely related sequence types suggests that these may confer a functional advantage to this lineage. Future work should aim to decipher the impact of these bacteriophages on the *S. pseudintermedius* lineages that encode them, in the context of pathogenicity and evolution.

We have observed differences between closely related genotypes in the replicon type – chromosome or plasmids – of specific genes conferring resistance to macrolides, trimethoprim and tetracycline, highlighting differences in genomic architecture. Plasmids encoding resistance determinants can be expected to be favoured by bacterial cells over chromosomally encoded resistance genes if the costs of maintenance and expression are lower [47,48]. Plasmid-mediated resistance genes have a higher proclivity for intraspecies and interspecies transfer, which has been observed in *Staphylococcus* [49], than those encoded on chromosomes. Results presented here indicate that the capability to encode a macrolide, trimethoprim or tetracycline resistance gene on the chromosome, plasmid or both is linked to sequence type and that these predicted plasmids are restricted to permissible genetic backgrounds. The sequence types that encode resistance genes on plasmids are likely to be responsible for the lateral, interspecies transfer of antibiotic resistance genes.

Given that these resistance genes are predicted to be plasmid-encoded, they may be present at a higher copy number than their chromosomal counterparts in other *S. pseudintermedius* lineages, and thereby confer higher levels of resistance to antibiotics used in the treatment of canine pyoderma and surgical-site infections. Distribution and genetic association of plasmid-encoded resistance genes is therefore likely to impact treatment efficacy.

This study has identified that mobile genetic elements, such as prophages and plasmids, are the primary determinants of diversification within *S. pseudintermedius*, consistent with previous studies [50,51]. Considering the association of prophages with lineages most frequently implicated in disease [51], a greater understanding of the impact of prophage genes and integration sites on *S. pseudintermedius* biology is required.

## supplementary material

10.1099/mgen.0.001369 Uncited Fig. S1.

10.1099/mgen.0.001369 Uncited Table S1.

## Abbreviations

HGT: horizontal gene transfer
MDR: multidrug-resistant
MRSP: methicillin-resistant *Staphylococcu pseudintermedius*
RM: restriction-modification
