# Supplementary material for: Global phylogenomic analysis of Staphylococcus pseudintermedius reveals genomic and prophage diversity in multidrug-resistant lineages
Source: Microb Genom. 2025 Mar 5;11(3):001369. doi: 10.1099/mgen.0.001369 (PMC11883136; doi:10.1099/mgen.0.001369)
Supplement: Uncited Fig. S1. [file mgen-11-01369-s001.pdf]

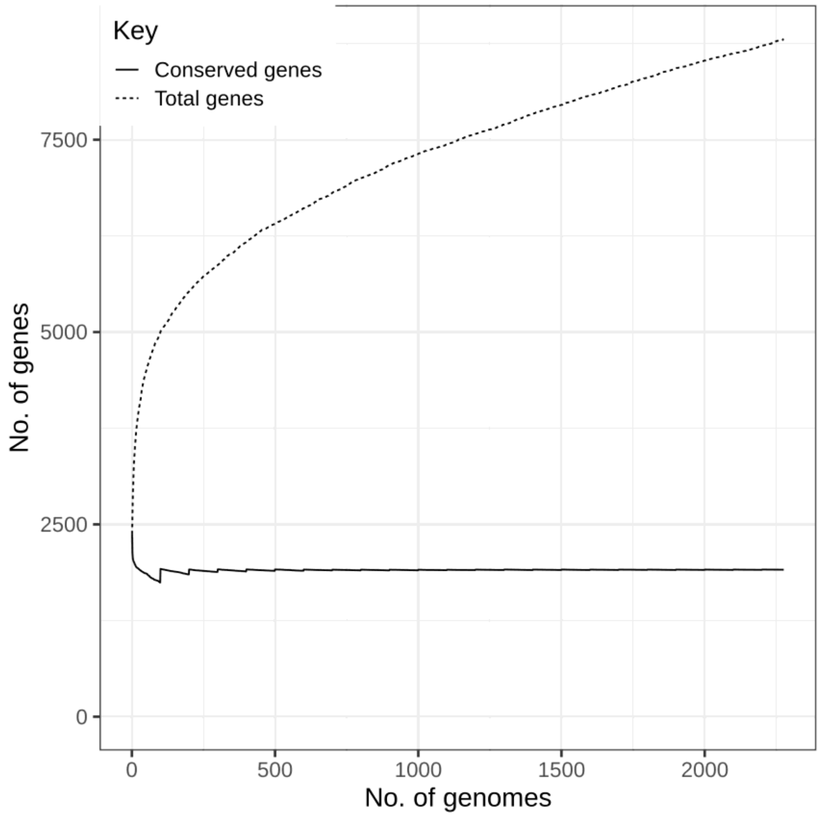

Figure S1: Gene accumulation curve. Analysis of the pangenome indicates the number of genes in the pangenome continues to increase with each additionally sequenced *S. pseudintermedius* genome included in the pangenome. Of the 6,814 accessory genes identified, 6,099 were only present 0-15% of genomes.

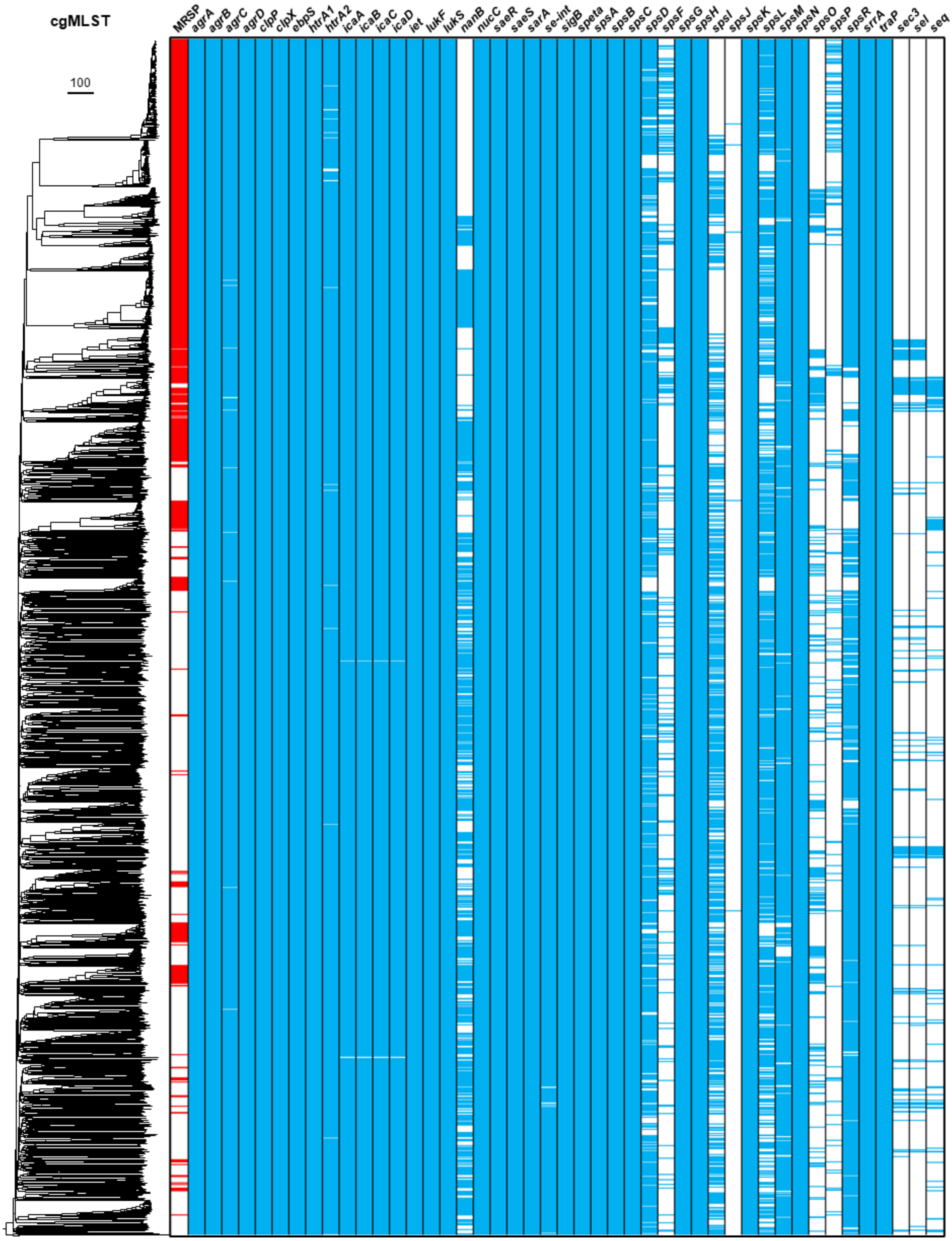

Figure S2: Maximum likelihood phylogeny showing the distribution of putative and reported virulence genes encoded by all 2,276 *S. pseudintermedius* genomes included in this study. Presence of a virulence gene is indicated by blue shading, absence is indicated by white shading. Full details of the phylogenetic distribution of virulence determinants are provided in Supplementary File 1.

Global phylogenomic analysis of Staphylococcus pseudintermedius reveals genomic and prophage diversity in multi-drug resistant lineages-Supplementary Material

A

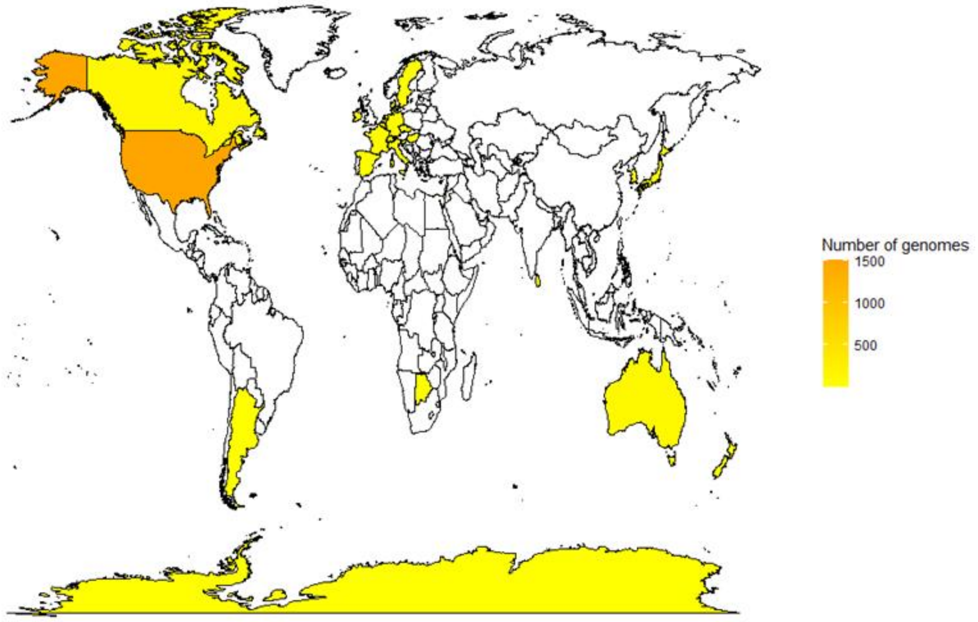

B

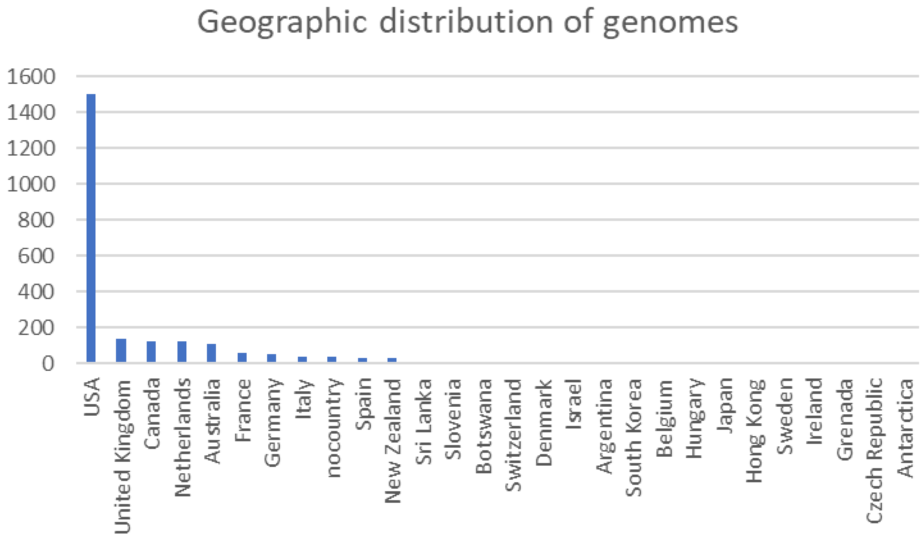

Figure S3: The geographic origin of isolates whose genome sequences were included in this study. **A**. Greater heat colour intensity indicates a greater number of genomes derived from that country. Most of the genomes included in this study are derived from USA. **B**. Bar chart showing the number of genomes derived from each country. Full details of the country of origin of each genome are provided in Supplementary File 1.

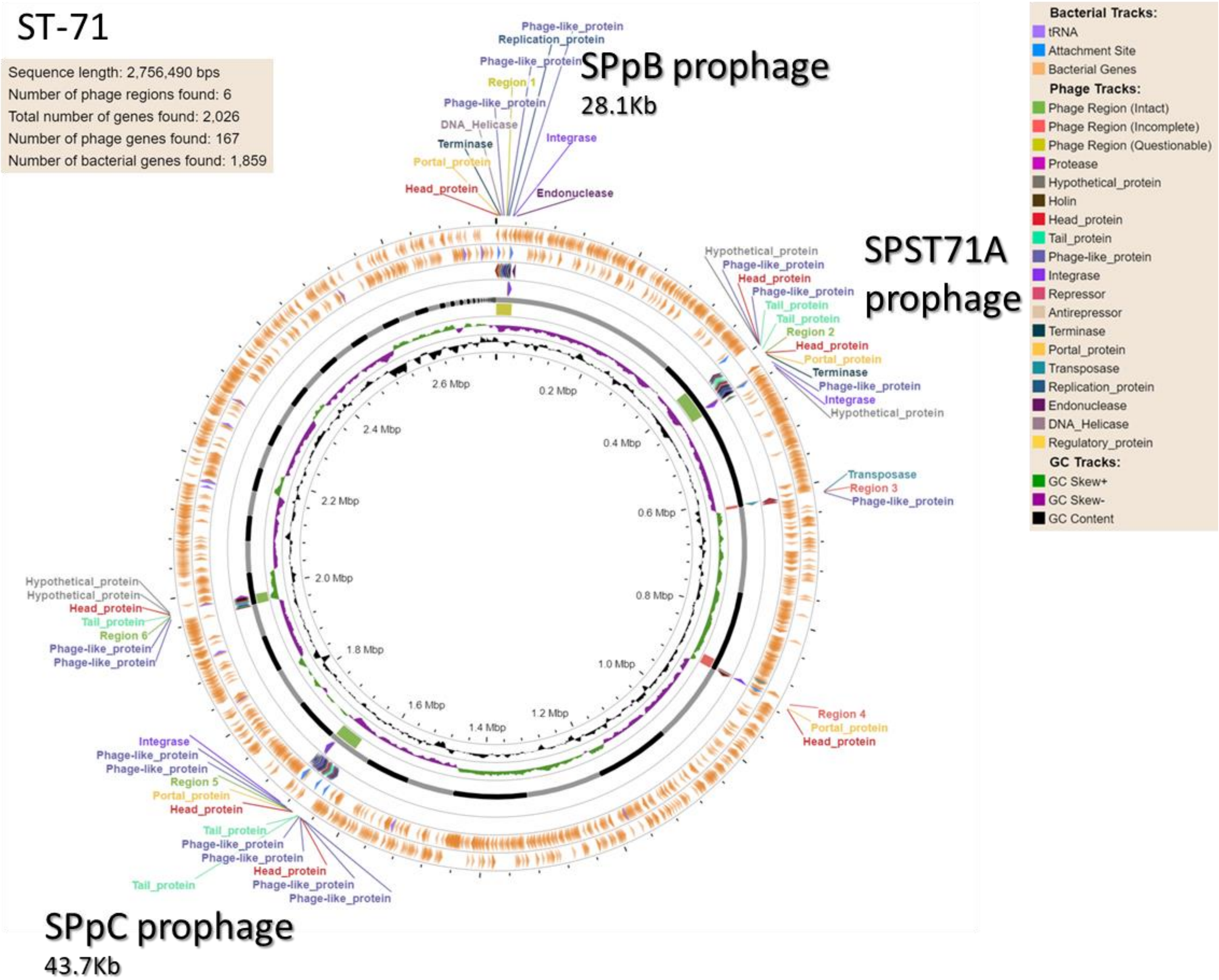

Figure S4: Circular genome map generated by PHASTEST, showing the number and location of intact prophages in a representative ST-71 genome (StaphpseudUoS10). Three large prophages SPST71A, SPpB, and SPpC have been identified in most ST-71 genomes which are absent from most other lineages.

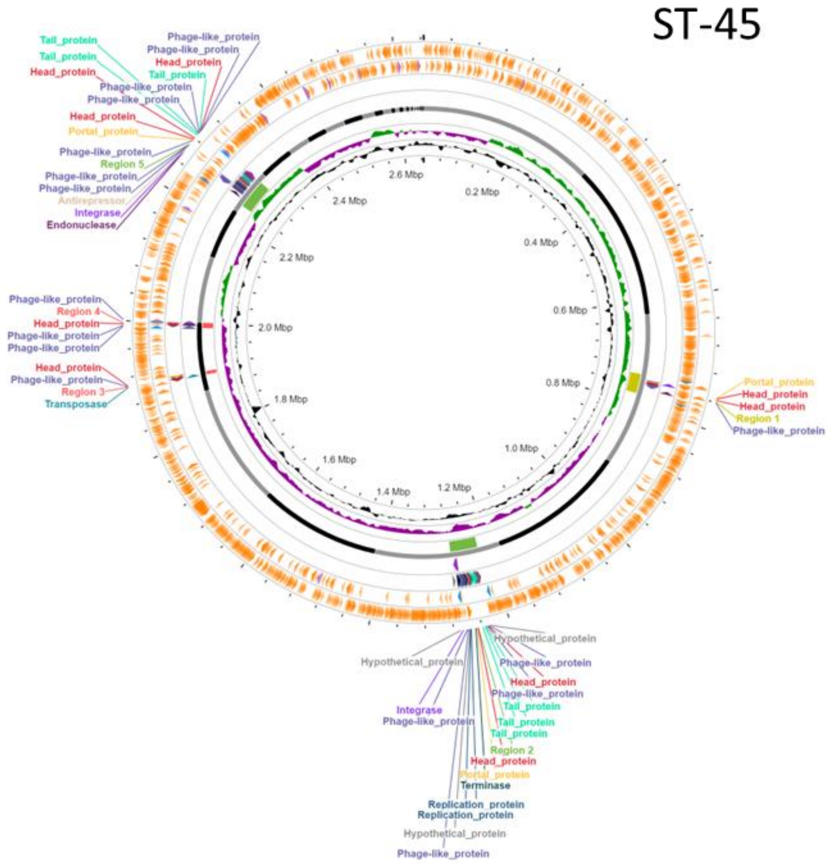

Figure S5: Circular genome map generated by PHASTEST, showing the number and location of intact prophages in a representative ST-45 genome. Most ST-45 genomes have at least 3 intact prophages, distinct from SPST71A, SPpB, and SPpC in ST-71.

Global phylogenomic analysis of Staphylococcus pseudintermedius reveals genomic and prophage diversity in multi-drug resistant lineages-Supplementary Material

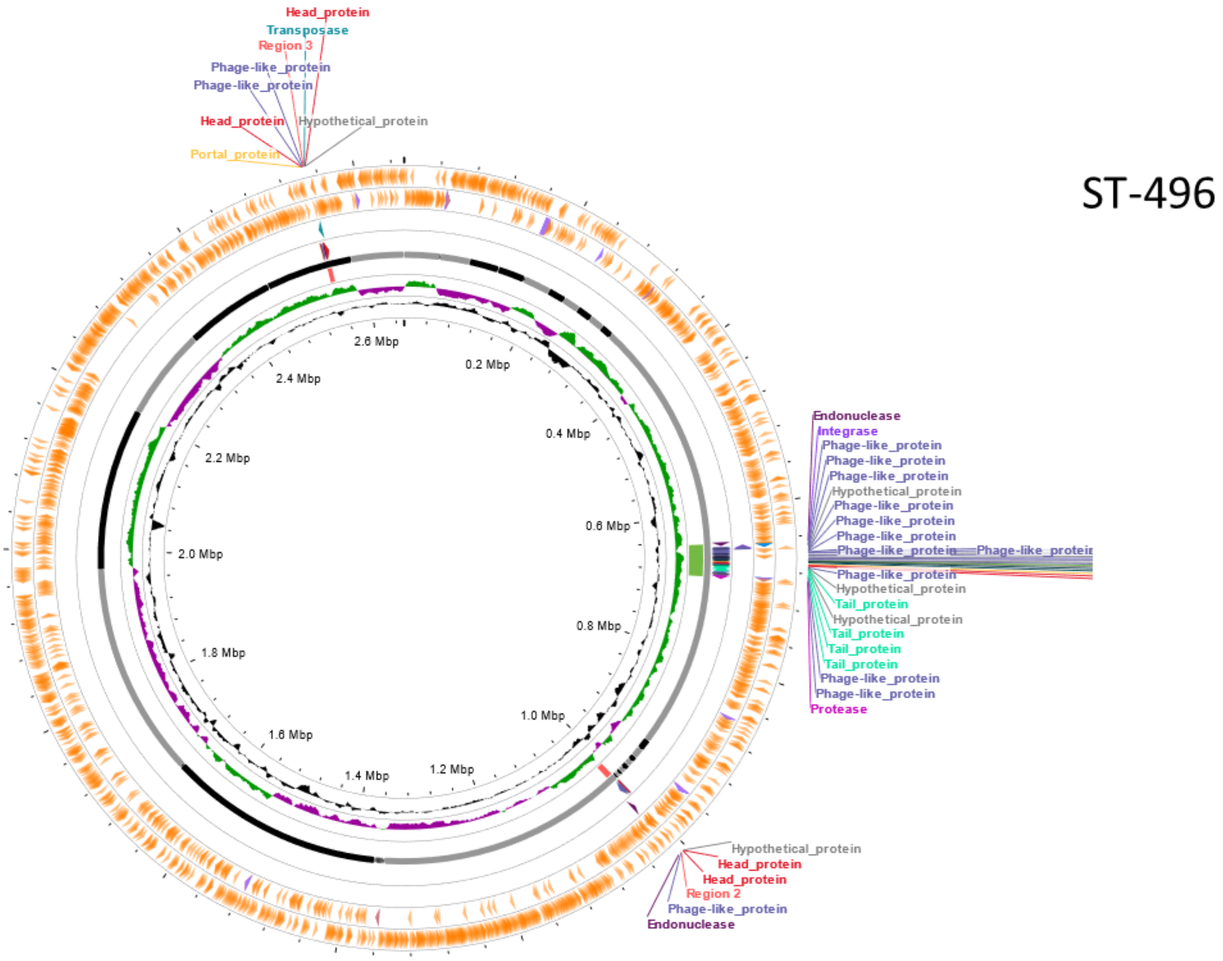

Figure S6: Circular genome map generated by PHASTEST, showing the number and location of intact prophages in a representative ST-496 genome. ST-496 only encode a single intact prophage.

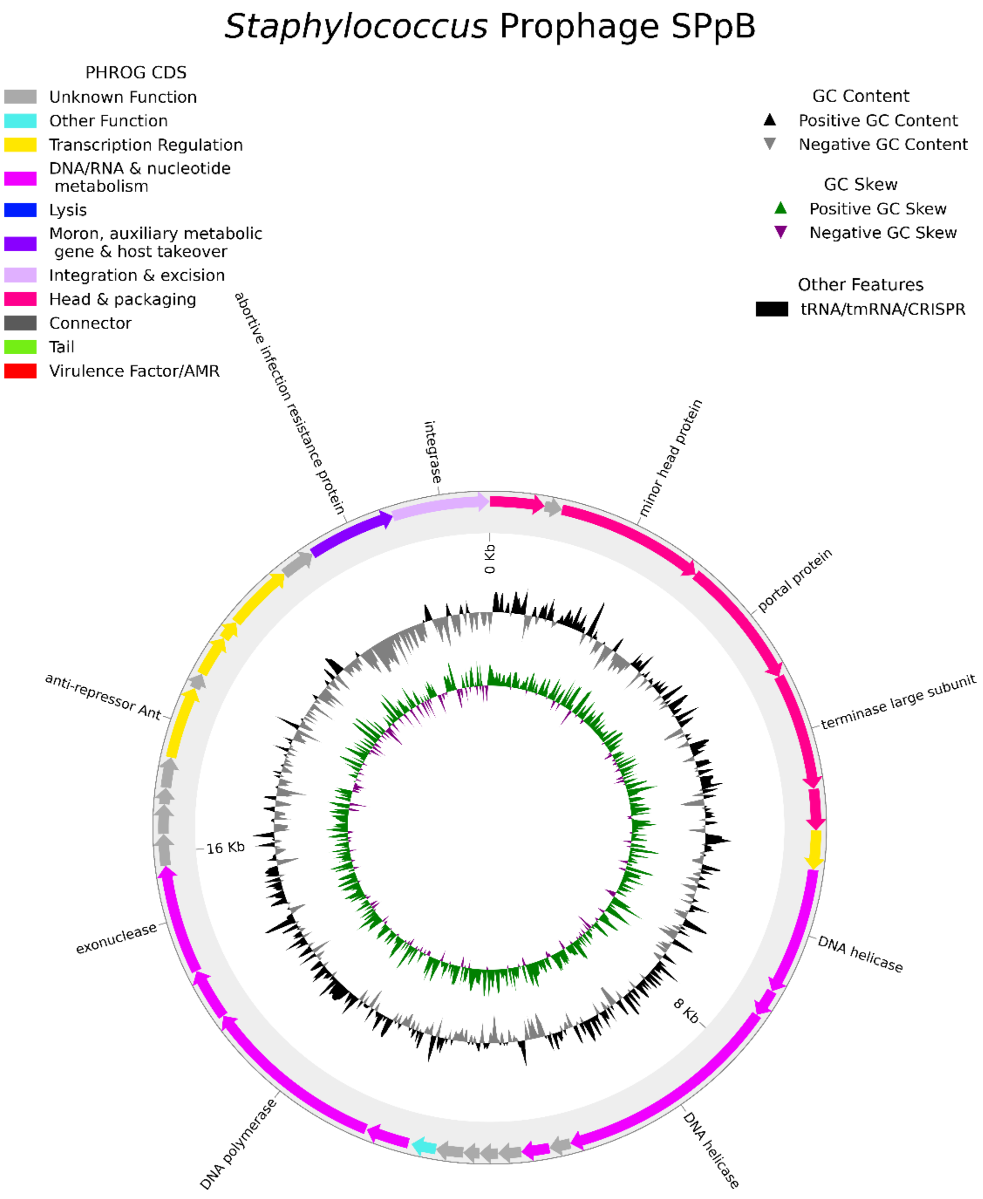

Figure S7: Schematic of the 28.1Kb SPpB prophage that is chromosomally integrated into ST-71, and in ST-45.

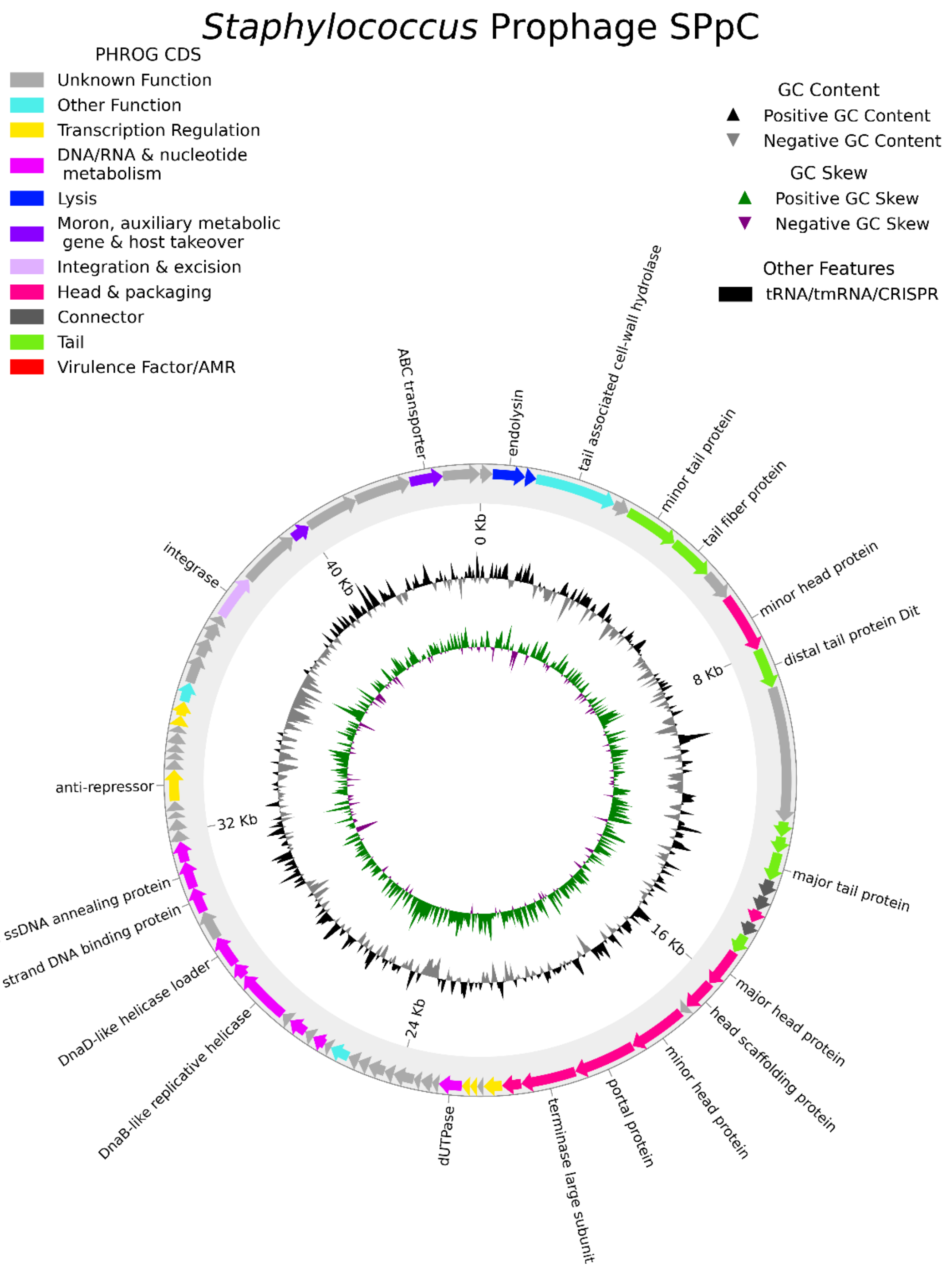

Figure S8: Schematic of the 43.7kb complete prophage associated with certain MRSP backgrounds.

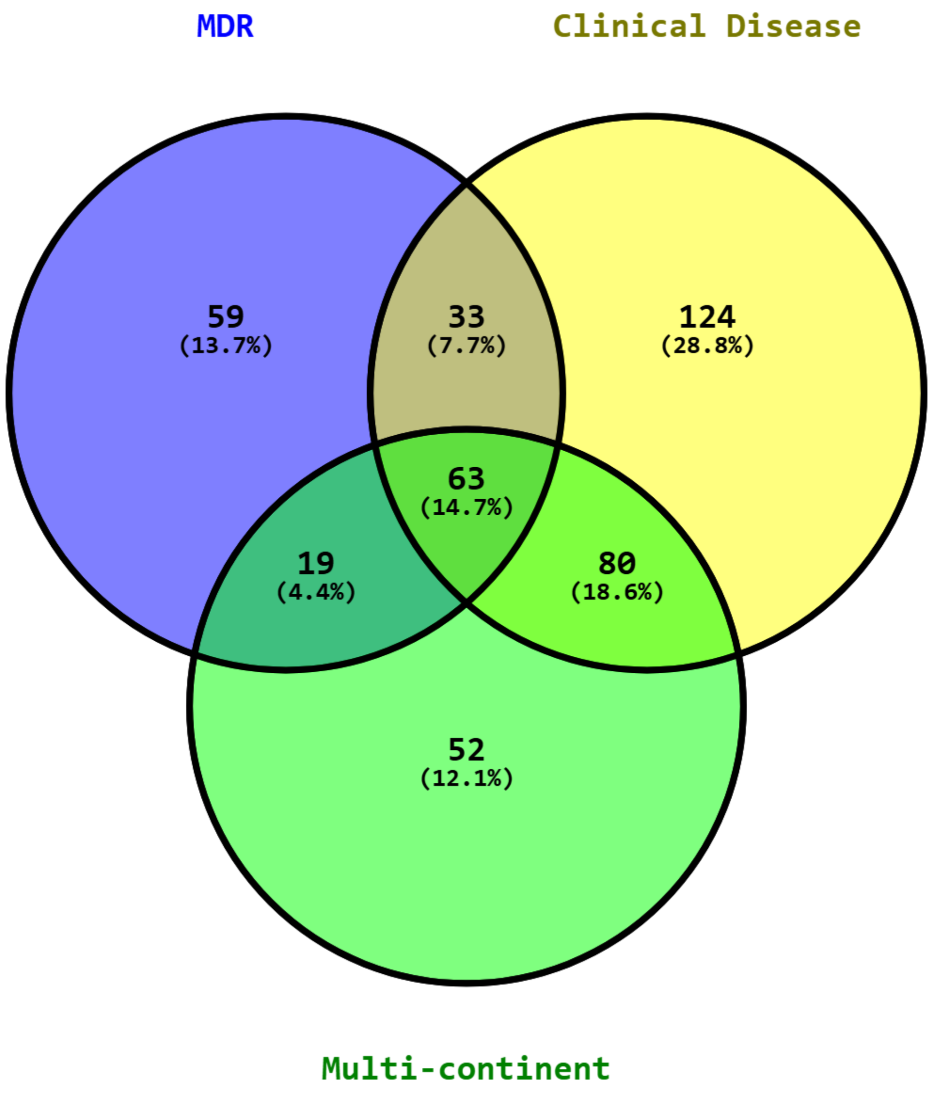

Figure S9: Venn diagram of genes associated genomes classified as “MDR”, associated with Clinical disease, and associated with multi-continent dissemination.

Global phylogenomic analysis of Staphylococcus pseudintermedius reveals genomic and prophage diversity in multi-drug resistant lineages-Supplementary Material

Table S1: List of 430 gene identified from GWAS as being over-represented in MDR, Clinical disease, and/or multi-continent dissemination. Putative phage genes are coloured in red font.

Gene	Annotation
ant(6)	aminoglycoside 6-adenylyltransferase
cat	Chloramphenicol acetyltransferase
dfrG	trimethoprim-resistant dihydrofolate reductase DfrG
ettA	Energy-dependent translational throttle protein EttA
group_1004	MFS transporter
group_1008	hypothetical protein
group_1013	hypothetical protein
group_1023	hypothetical protein
group_1028	APH(3') family aminoglycoside O-phosphotransferase
group_1044	hypothetical protein
group_1048	Pin-related site-specific recombinase/DNA invertase
group_1068	CRISPR repeat RNA endoribonuclease Cas6
group_1095	hypothetical protein
group_1098	aminotransferase class V
group_111	cytochrome
group_1118	hypothetical protein
group_1137	DNA polymerase
group_1138	nuclease
group_1147	repE protein
group_1149	peptide ABC transporter ATP-binding protein
group_1161	hypothetical protein
group_1164	transcriptional regulator
group_1167	isochorismatase
group_1175	hypothetical protein
group_1178	glycerophosphoryl diester phosphodiesterase
group_1179	hypothetical protein
group_118	Tricarboxylate transport protein TctC
group_1182	methyltransferase
group_1185	hypothetical protein
group_120	recombinase RecB
group_1211	membrane protein, putative
group_1212	hypothetical protein
group_1244	hypothetical protein
group_1247	hypothetical protein
group_1249	hypothetical protein
group_127	aminoglycoside phosphotransferase APH(3')
group_1295	hypothetical protein
group_1296	hydrolase
group_1312	hypothetical protein
group_1313	BetR domain protein
group_1336	hypothetical protein
group_1338	DEAD/DEAH box helicase
group_1339	nuclease
group_134	putative cell-wall-anchored protein SasA (LPXTG motif)
group_1349	hypothetical protein
group_1350	hypothetical protein
group_1352	IS256 family transposase IS256

group_1368	Phage protein
group_1370	holin
group_1371	hypothetical protein
group_1383	transcriptional regulator
group_1385	peptide ABC transporter ATP-binding protein
group_1386	peptide ABC transporter permease
group_139	Teichoic acid biosynthesis protein
group_1392	YxeA family protein
group_1393	hypothetical protein
group_1416	Integrase
group_1417	hypothetical protein
group_143	toxin
group_1448	nucleotidyltransferase domain-containing protein
group_145	melibiose carrier protein
group_1452	CRISPR-associated protein, Csm5 family
group_1454	hypothetical protein
group_1482	putative acyl dehydratase MaoC
group_1527	hypothetical protein
group_1528	hypothetical protein
group_1529	DnaD domain protein
group_1530	DNA replication protein DnaC
group_1531	terminase
group_1532	peptidase
group_1533	phage gp6-like head-tail connector protein
group_154	hypothetical protein
group_1552	Transcriptional regulator, GntR family
group_1553	hypothetical protein
group_1557	bacteriocin-associated protein
group_1585	N-acetyltransferase
group_1586	Insertion sequence IS5376 putative ATP-binding protein
group_160	peptidase
group_1617	YncE family protein
group_1632	hypothetical protein
group_1646	recombinase family protein
group_1649	hypothetical protein
group_1652	hypothetical protein
group_1653	poly(glycerol-phosphate) alpha-glucosyltransferase
group_1658	hypothetical protein
group_166	ATPase
group_1677	phage capsid protein
group_1679	hypothetical protein
group_1684	hypothetical protein
group_1719	Sporulation initiation inhibitor protein Soj
group_1740	integrase
group_1753	Putative glycosyl/glycerophosphate transferases involved in teichoic acid biosynthesis TagF/TagB/EpsJ/RodC
group_179	hypothetical protein
group_181	sigma-70 family RNA polymerase sigma factor
group_1828	hypothetical protein
group_1830	hypothetical protein
group_1843	hypothetical protein
group_1844	SGNH/GDSL hydrolase family protein
group_1855	hypothetical protein

group_1861	MFS transporter
group_1881	hypothetical protein
group_1882	hypothetical protein
group_1883	Rha family transcriptional regulator
group_1884	hypothetical protein
group_1898	hypothetical protein
group_191	transposase
group_1918	serine protease
group_1919	Phage minor structural protein
group_192	hypothetical protein
group_1920	Phage minor structural protein
group_1921	phage tail protein
group_1935	hypothetical protein
group_1940	hypothetical protein
group_1942	MBL fold metallo-hydrolase
group_195	peptide ABC transporter substrate-binding protein
group_1954	hypothetical protein
group_197	Phage tail length tape-measure protein
group_1971	hypothetical protein
group_1980	hypothetical protein
group_1985	Streptothricin acetyltransferase
group_199	tRNA-dependent cyclodipeptide synthase
group_20	YSIRK signal domain/LPXTG anchor domain surface protein
group_2035	CRISPR-associated protein, Csm2 family
group_2036	CRISPR-associated RAMP protein, Csm4 family
group_2038	hypothetical protein
group_206	MFS transporter
group_2139	hypothetical protein
group_218	hypothetical protein
group_2180	NUDIX hydrolase
group_2194	phage portal protein
group_2195	Phage minor structural protein
group_2196	hypothetical protein
group_2216	hypothetical protein
group_2244	Phage protein
group_2245	Phage protein
group_2257	hypothetical protein
group_2263	peptide-binding protein
group_2286	Phage protein
group_2302	siphovirus Gp157 family protein
group_2313	hypothetical protein
group_2316	mecA-type methicillin resistance repressor MecI
group_2317	dihydroneopterin aldolase
group_2318	hypothetical protein
group_2324	hypothetical protein
group_2325	transcriptional regulator
group_2333	hypothetical protein
group_236	hypothetical protein
group_2376	hypothetical protein
group_2379	transcriptional repressor
group_2391	membrane protein
group_240	recombinase
group_2451	PhnB protein; putative DNA binding 3- demethylubiquinone-9 3-methyltransferase domain protein

group_2452	hypothetical protein
group_2453	hypothetical protein
group_246	terminase
group_2470	hypothetical protein
group_254	terminase
group_258	holin
group_2618	hypothetical protein
group_2624	DMT family transporter
group_2647	DNA-binding protein
group_2648	XRE family transcriptional regulator
group_2649	hypothetical protein
group_2650	Phage protein
group_2651	hypothetical protein
group_2652	hypothetical protein
group_2653	hypothetical protein
group_2665	hypothetical protein
group_2681	transcriptional regulator
group_2722	hypothetical protein
group_2723	hypothetical protein
group_2724	Phage DNA binding protein
group_2725	hypothetical protein
group_2726	transcriptional regulator
group_2727	hypothetical protein
group_2737	hypothetical protein
group_2746	sugar ABC transporter permease
group_2756	LSM domain protein
group_2761	Phage protein
group_2766	hypothetical protein
group_2768	phage tail protein
group_2769	Phage protein
group_278	hypothetical protein
group_2784	putative cell-wall-anchored protein SasA (LPXTG motif)
group_2786	hypothetical protein
group_2788	hypothetical protein
group_2795	hypothetical protein
group_2796	hypothetical protein
group_2803	cytoplasmic protein
group_2804	hypothetical protein
group_2805	ACP synthase
group_2806	ADP-ribosyltransferase
group_2807	hypothetical protein
group_2820	hypothetical protein
group_2825	hypothetical protein
group_285	hypothetical protein
group_2850	hypothetical protein
group_2851	Antirestriction protein
group_2852	hypothetical protein
group_2922	hypothetical protein
group_2927	transposon DNA-invertase
group_2928	hypothetical protein
group_2929	hypothetical protein
group_2930	hypothetical protein
group_2931	hypothetical protein

group_2946	CRISPR-associated RAMP Csm3
group_2969	Antitoxin epsilon
group_297	restriction endonuclease subunit S
group_299	hypothetical protein
group_302	Tetracycline resistance protein TetM
group_303	hypothetical protein
group_3037	intracellular adhesion protein D
group_305	transposase
group_3127	hypothetical protein
group_3143	hypothetical protein
group_3159	hypothetical protein
group_316	hypothetical protein
group_317	hypothetical protein
group_32	BlaR1 family beta-lactam sensor/signal transducer
group_321	transposase
group_3212	hypothetical protein
group_3216	hypothetical protein
group_3233	transcriptional regulator
group_3234	hypothetical protein
group_3235	phage capsid protein
group_3236	hypothetical protein
group_3237	phage tail protein
group_3238	hypothetical protein
group_3239	Phage minor structural protein
group_3240	hypothetical protein
group_3241	chromosome partitioning protein ParB
group_326	Poly(glycerol-phosphate) alpha- glucosyltransferase
group_3262	hypothetical protein
group_3296	Phage protein
group_33	Protein A, von Willebrand factor binding protein Spa
group_331	6-phospho-beta-glucosidase
group_3312	hypothetical protein
group_3315	23S rRNA methyltransferase
group_3316	hypothetical protein
group_3318	LSM domain protein
group_3337	hypothetical protein
group_3339	hypothetical protein
group_334	ABC transporter ATP-binding protein
group_3343	hypothetical protein
group_3344	hypothetical protein
group_3345	XRE family transcriptional regulator
group_3346	phage major tail protein, TP901-1 family
group_3347	hypothetical protein
group_336	ABC transporter ATP-binding protein
group_3365	signal transduction protein TRAP
group_3366	hypothetical protein
group_3367	hypothetical protein
group_3369	hypothetical protein
group_338	glutamyl endopeptidase
group_3392	hypothetical protein
group_3393	hypothetical protein
group_3400	single-stranded DNA-binding protein
group_3407	tripartite tricarboxylate transporter TctB family protein

group_344	hypothetical protein
group_3464	hypothetical protein
group_3465	hypothetical protein
group_3466	hypothetical protein
group_3467	hypothetical protein
group_3488	hypothetical protein
group_350	hypothetical protein
group_352	hypothetical protein
group_355	hypothetical protein
group_3570	hypothetical protein
group_3571	hypothetical protein
group_3572	hypothetical protein
group_3573	hypothetical protein
group_3595	hypothetical protein
group_3599	hypothetical protein
group_36	Type II restriction modification system
group_3602	CRISPR-associated endoribonuclease Cas2
group_3607	hypothetical protein
group_3608	Type I restriction-modification system, restriction subunit R
group_3627	hypothetical protein
group_3636	Pin-related site-specific recombinase/DNA invertase
group_3637	Protein adenylyltransferase NmFic
group_3638	IS30 family transposase IS1252
group_369	hypothetical protein
group_375	glycosyltransferase family 2 protein
group_386	PE_PGRS (wag22)
group_3864	hypothetical protein
group_387	holin
group_392	hypothetical protein
group_3990	hypothetical protein
group_3991	hypothetical protein
group_3992	hypothetical protein
group_407	hypothetical protein
group_409	CRISPR-associated protein, Csm1 family
group_4125	hypothetical protein
group_415	membrane protein
group_421	hypothetical protein
group_4238	hypothetical protein
group_4239	transposase
group_4243	hypothetical protein
group_4244	sigma-70 family RNA polymerase sigma factor
group_4245	alpha/beta hydrolase
group_4248	hypothetical protein
group_4249	hypothetical protein
group_4250	hypothetical protein
group_4253	hypothetical protein
group_4254	hypothetical protein
group_4255	hypothetical protein
group_4259	hypothetical protein
group_4260	bacteriocin transporter
group_4274	hypothetical protein
group_4275	hypothetical protein
group_4276	hypothetical protein

group_4277	hypothetical protein
group_4287	hypothetical protein
group_4288	peptide ABC transporter permease
group_429	CDP-glycerol--glycerophosphate glycerophosphotransferase
group_4290	hypothetical protein
group_4305	Phage protein
group_4316	hypothetical protein
group_4317	hypothetical protein
group_4318	hypothetical protein
group_4319	hypothetical protein
group_432	ABC transporter substrate-binding protein
group_4336	rhodanese
group_4337	hypothetical protein
group_4338	hypothetical protein
group_4339	hypothetical protein
group_434	Phage minor structural protein
group_4340	recombinase RecA
group_4341	hypothetical protein
group_4343	TIR domain-containing protein
group_4344	hypothetical protein
group_4346	riboflavin biosynthesis protein RibD
group_4347	WYL domain-containing protein
group_4348	hypothetical protein
group_435	Acetyltransferase
group_4427	Xis-Tn protein
group_4428	Conjugation related protein
group_4433	hypothetical protein
group_4434	hypothetical protein
group_4446	hypothetical protein
group_4479	oxidoreductase
group_4583	putative primase
group_4584	hypothetical protein
group_4609	hypothetical protein
group_4610	hypothetical protein
group_4613	hypothetical protein
group_4614	hypothetical protein
group_4615	hypothetical protein
group_4693	hypothetical protein
group_4695	replication protein
group_4696	Toxin zeta
group_4697	peptide-binding protein
group_477	arginine--tRNA ligase
group_487	DNA repair helicase
group_49	trypsin
group_4914	hypothetical protein
group_512	glutamyl endopeptidase
group_521	ATP-binding protein
group_539	hypothetical protein
group_54	transposase
group_540	hypothetical protein
group_553	YIP1 family protein
group_56	bacteriocin-associated protein
group_568	diguanylate cyclase/phosphodiesterase (GGDEF & EAL domains) with PAS/PAC sensor(s)

group_5898	hypothetical protein
group_597	transcriptional regulator
group_601	DNA helicase
group_635	Transcriptional regulator, DeoR family
group_646	CRISPR-associated protein Csm6
group_652	MBL fold metallo-hydrolase
group_653	hypothetical protein
group_657	hypothetical protein
group_664	MFS transporter
group_681	DEAD/DEAH box helicase
group_686	Phage terminase, small subunit
group_688	hypothetical protein
group_691	hypothetical protein
group_719	putative primase
group_72	permease
group_733	hypothetical protein
group_74	hypothetical protein
group_751	transposon DNA-invertase
group_761	hypothetical protein
group_775	nucleoid-structuring protein H-NS
group_776	methicillin resistance protein
group_778	N-6 DNA methylase
group_781	hypothetical protein
group_782	hypothetical protein
group_785	hypothetical protein
group_81	MutR family transcriptional regulator
group_82	hypothetical protein
group_826	MarR family transcriptional regulator
group_834	hypothetical protein
group_8342	RNA methyltransferase
group_8377	hypothetical protein
group_8433	peptide ABC transporter permease
group_845	single-stranded DNA-binding protein
group_8542	GNAT family N-acetyltransferase
group_8554	dihydrofolate reductase
group_86	Acetyltransferase, GNAT family
group_869	gfo/Idh/MocA family oxidoreductase
group_8743	alpha/beta hydrolase
group_877	hypothetical protein
group_882	hypothetical protein
group_889	hypothetical protein
group_896	NAD-dependent dehydratase
group_897	integrase
group_920	ROK family transcriptional regulator
group_924	CRISPR-associated protein Cas1
group_936	hypothetical protein
group_94	glutamyl endopeptidase
group_961	site-specific integrase
group_963	Phage protein
group_974	hypothetical protein
group_979	hypothetical protein
group_98	ATP-dependent DNA helicase RecQ
group_996	hypothetical protein

htpX	hypothetical protein
mdtL	MFS transporter
mecA	PBP2a family beta-lactam-resistant peptidoglycan transpeptidase MecA
murF	UDP-N-acetylmuramoylalanyl-D-glutamyl-2,6- diaminopimelate--D-alanyl-D-alanine ligase
pknD	hypothetical protein
rbsD	hypothetical protein
repD	replication initiation protein
repN	Replication initiation protein
rimJ	hypothetical protein
rsmA	23S rRNA (adenine(2058)-N(6))-methyltransferase Erm(B)
rutB	isochorismatase
soj	Chromosome-partitioning ATPase Soj
topA2	DNA topoisomerase

Table S2:

**Genes associated with Multi-Drug resistance**	**Genes associated with multi-continent dissemination**	**Genes associated with Clinical disease genomes**
group_1028	group_1118	group_317
group_1182	group_681	group_1118
group_1383	group_778	group_877
group_1985	group_350	group_2316
ant(6)	group_2737	group_1179
group_1448	group_297	group_2805
rsmA	group_98	group_2806
group_2263	rimJ	group_285
topA2	group_392	group_4697
group_1898	group_1179	group_98
dfrG	group_776	group_597
group_321	group_2324	group_254
group_568	group_181	group_246
group_4287	group_1417	group_1920
group_1048	group_139	group_1371
group_1147	group_118	group_1919
group_4346	group_3407	group_1921
group_3400	group_688	group_434
group_86	group_429	group_134
group_3316	group_3234	group_197
group_4347	group_375	group_3346
group_127	group_4254	group_2286
group_1178	group_4255	group_2768
mecA	group_1249	group_2769
group_1482	group_2038	group_3347
group_775	group_540	group_4305
group_3315	group_1954	group_1137
group_1211	group_326	group_3296
group_1212	group_1753	group_1138

group_1416	group_2453	group_1338
group_1980	group_36	group_1881
group_2850	group_120	group_1339
group_2851	group_896	group_316
group_2852	group_1529	group_1882
group_3159	group_719	group_2725
group_3466	group_3595	group_3312
group_4427	group_4584	group_776
group_3464	group_2946	group_2313
group_3467	group_3608	group_2723
group_4428	group_4613	group_1585
group_2139	group_4615	group_2244
group_3465	group_1452	group_4275
group_2257	group_3602	group_4276
group_1352	group_409	group_2724
group_4446	group_4583	group_2245
group_120	group_2036	group_2746
group_4583	group_2035	group_2803
group_3595	group_2727	group_920
group_4584	group_302	group_2470
group_2946	group_782	group_4277
group_3608	group_924	soj
group_4613	group_4340	group_2969
group_4615	group_4914	group_1370
group_1452	group_1884	group_3345
group_3602	group_4614	group_1677
group_409	group_2318	group_421
group_2036	group_4341	group_4336
group_1454	group_1646	group_4337
group_2035	group_3393	group_4338
group_924	group_2804	group_2318
group_4614	group_4339	group_4341
group_3599	group_961	group_2756
group_4609	group_3599	group_297
group_751	group_4337	group_192
group_1247	group_4338	group_278
group_2451	group_4609	group_4274
group_1068	group_1312	group_415
group_305	group_2647	group_302
group_2453	group_3233	group_2216
group_407	group_1247	group_3636
group_719	group_996	group_3637
group_1971	group_1528	group_4254
group_3216	group_2376	group_2317
group_236	group_845	group_1942
group_1249	group_1313	group_1646

group_2825	group_1454	group_3393
group_2038	group_1068	group_2804
group_521	group_407	group_4339
group_302	group_1843	group_4340
group_2180	group_4243	group_4914
group_1295	group_2196	group_996
group_4239	group_3240	group_2649
group_4259	group_3241	group_1529
group_635	group_4249	group_4244
group_4610	group_4250	group_1095
group_2624	group_4248	group_1417
group_1296	group_1677	group_4693
group_974	group_336	group_539
group_646	group_2451	group_1211
repD	group_4274	group_1212
group_1098	group_4277	group_1416
cat	group_2313	group_1980
group_32	group_2805	group_2850
group_3337	group_2806	group_2851
group_3339	group_2245	group_2852
group_3636	group_1971	group_3159
group_3637	group_2648	group_3466
group_2766	group_1175	group_4427
group_882	group_1855	group_3467
soj	group_166	group_4428
group_2969	group_882	group_2139
group_2470	group_2766	group_1185
group_36	group_3392	group_785
group_3638	group_3607	group_691
group_4253	group_236	group_392
group_1617	group_1679	rimJ
group_4697	group_2796	group_681
group_4693	group_1942	group_4255
group_3343	group_657	group_350
group_3262	group_355	group_1679
group_3344	group_686	group_2650
group_2391	group_2317	group_2726
group_4479	group_2452	group_4245
group_2618	group_4610	group_3234
group_2216	group_81	group_3464
group_355	group_4336	group_664
murF	group_635	group_1828
group_1740	group_1023	group_3212
group_4348	group_1370	group_4238
group_4695	group_1586	group_2722
group_1653	group_2803	group_778

group_1918	group_920	group_781
group_785	group_154	group_2324
group_1004	group_963	group_1527
group_415	group_1940	group_2665
group_782	group_74	group_1531
group_54	group_1530	group_1336
mdtL	group_652	group_3465
group_3627	htpX	group_4446
rutB	group_1918	group_3392
group_435	group_646	group_20
group_303	group_2650	group_2653
group_1167	group_653	pknD
group_1185	group_2795	group_3488
group_1828	group_2316	group_1244
group_206	group_1531	group_1830
group_3318	group_4244	group_3638
group_3607	group_240	group_432
group_33	group_2653	group_195
group_3212	pknD	group_4288
group_4238	group_3345	group_1149
group_1553	group_4245	group_1349
group_1652	group_601	repN
group_2325	group_72	group_4434
group_3312	group_664	group_4433
pknD	group_369	group_3864
group_1008	group_1861	group_181
group_4696	group_334	group_897
group_2452	group_3312	group_2737
group_487	group_979	group_352
group_553	group_1211	group_1048
group_2316	group_1212	group_1147
group_2653	group_1416	group_4287
group_299	group_1980	group_82
group_2317	group_2850	group_8342
group_826	group_2851	group_782
group_1942	group_2852	group_2786
group_4336	group_3159	group_1649
group_2803	group_3466	group_4344
group_920	group_4427	group_429
group_1684	group_4428	group_2927
group_199	group_3464	group_1719
group_1557	group_3467	group_3573
group_2922	group_2139	group_2930
group_317	group_3465	group_2931
group_56	group_1585	group_3127
group_4340	group_3212	group_2928

group_4914	group_4238	group_3570
group_1392	group_2649	group_3571
group_1585	group_4260	group_3572
group_2318	group_1385	group_2929
group_4341	group_317	group_540
group_2681	group_1149	group_2820
group_3037	group_1386	group_1393
	group_160	group_139
	group_2379	group_118
	group_8542	group_3407
	group_761	group_326
	group_8554	group_1753
	group_49	group_375
	group_94	group_896
	group_218	group_688
	group_145	group_4343
	group_869	group_1884
	group_331	group_36
	group_191	group_2453
	group_1552	group_1249
	ettA	group_2727
	group_1392	group_2038
	group_56	group_1954
	group_344	group_3595
	group_8377	group_4584
	group_86	group_143
	group_8743	group_2946
	group_512	group_3608
	group_936	group_4613
	group_338	group_4615
	group_179	group_409
	group_889	group_1843
	group_1935	group_1452
	rbsD	group_3602
	group_1632	group_120
	group_2784	group_1855
	group_386	group_2035
	group_387	group_1313
	group_1013	group_4614
	group_1028	group_3233
	group_1182	group_4243
	group_1985	group_4583
	group_1448	group_2036
	group_8433	group_1883
	group_1383	group_2196
	ant(6)	group_3240

topA2	group_3241
	group_4249
	group_4250
	group_4248
	group_1528
	group_2376
	group_845
	group_961
	group_1312
	group_2647
	group_3599
	group_924
	group_166
	group_2648
	group_4290
	group_1247
	group_407
	group_719
	group_1350
	group_1161
	group_4316
	group_1164
	group_3367
	group_4317
	group_477
	group_4125
	group_4609
	group_4319
	group_1068
	group_154
	group_206
	group_3369
	group_1454
	group_5898
	group_3990
	group_3992
	group_834
	group_733
	group_3991
	group_3143
	group_3366
	group_2807
	group_4318
	group_2302
	group_3337
	group_1918

	group_2761
	group_1586
	group_2788
	group_2766
	group_882
	group_974
	group_336
	group_4259
	group_111
	group_1658
	group_435
	group_4253
	group_1175
	group_3262
	group_1482
	group_2624
	mecA
	group_3365
	group_1178
	group_1368
	group_1296
	group_3216
	group_2333
	group_1004
	group_601
	group_1044
	group_775
	group_258
	group_2194
	group_2651
	group_2652
	group_3343
	group_1532
	group_1533
	group_1844
	group_2195
	group_3235
	group_3236
	group_3237
	group_3238
	group_3239
